# Modeling and optimization of sustainable ternary concrete incorporating rice husk ash and extracted micro silica

**DOI:** 10.1038/s41598-026-35983-8

**Published:** 2026-01-12

**Authors:** Muhammad Fahad Ullah, Hesheng Tang, Asad Ullah, Shoaib Ahmad, Abdullah Alzlfawi, Mahmood Ahmad, Zsolt Tóth

**Affiliations:** 1 Department of Disaster Mitigation for Structures, College of Civil Engineering, Tongji University, Shanghai, 200092, China; 2 Department of CS, Wing Head (Enabling Technology, CoE-AI), Bahria University, E8, Islamabad, Pakistan; 3 College of Civil Engineering, Tongji University, Shanghai, 200092 China; 4 Department of Civil and Environmental Engineering, Majmaah University, Al Majmaah, 11952, Saudi Arabia; 5 Department of Civil Engineering, University of Engineering and Technology (Bannu Campus), Bannu, 28100 Pakistan; 6 Institute of Energy Infrastructure, Universiti Tenaga Nasional, Kajang, 43000 Malaysia; 7 Faculty of Wood Engineering and Creative Industries, University of Sopron, Sopron, Hungary

**Keywords:** Artificial neural network (ANN), Compressive strength (CS), Extracted micro-silica (EMS), Optimization, Rice husk ash (RHA), Response surface methodology (RSM), Sustainable concrete, Engineering, Environmental sciences, Materials science

## Abstract

Concrete production accounts for a significant share of global CO_2_ emissions, underscoring the need for sustainable supplementary cementitious materials. This study evaluates a ternary cementitious system incorporating extracted micro-silica (EMS) and rice husk ash (RHA) as partial cement replacements to enhance compressive strength and reduce cement dependency. An experimental program was conducted on mixtures with varying EMS and RHA dosages, followed by predictive modelling and optimization using Response Surface Methodology (RSM) and Artificial Neural Networks (ANN). Optimal mixtures containing 10–15% EMS and 15–25% RHA achieved higher compressive strength than the control mix, whereas higher replacement levels reduced strength due to particle agglomeration and weak hydration products. SEM analysis confirmed the improved microstructure in the optimized mixture, characterized by refined C-S-H networks and reduced porosity. The RSM model achieved strong predictive accuracy (R² = 0.95, RMSE = 2.7 MPa), while the ANN model achieved R² = 0.98 and RMSE = 1.9 MPa. These findings provide valuable insights for designing high-performance, sustainable concrete that relies less on traditional cementitious materials. Future work should focus on evaluating the long-term durability and environmental impact of the optimized mixtures in real-world applications.

## Introduction

 Concrete is a vital, widely used manufactured material in the built environment, valued for its strength, durability, and cost-effectiveness. However, cement is a key component of concrete and a significant source of embodied carbon, accounting for approximately 7% of global carbon dioxide emissions from human activities^1,2^. Therefore, to mitigate the growing threats of climate change, it is essential to reduce the carbon footprint of cement production and minimize its environmental impact^3,4^. The International Energy Agency reports that direct CO_2_ emissions from cement production increased by 1.5% annually between 2015 and 2021, underscoring the dire need to incorporate alternative agro-waste materials as replacements for cement and concrete in manufacturing^5^. A UNEP-SBCI report identified supplementary cementitious materials (SCMs) as the most effective solution for reducing CO_2_ emissions in cement production, offering minimal economic or performance trade-offs^6^. Sustainable construction practices, such as replacing cement with high-reactivity SCMs, can reduce environmental impact while producing durable and eco-friendly concrete^7,8^. Extensive research has explored SCMs, including RHA^9^, steel slag^10^, metakaolin^11^, waste glass sludge^12^, Anacardium occidentale nutshell ash^13^, and micro-silica (MS)^14^, as sustainable cement alternatives. The need for novel, high-performance cementitious solutions is further underscored by recent advances in sustainable binders and advanced concrete modeling^15–17^. Recent studies highlight MS derived from RHA as a promising SCM. High pozzolanic reactivity and amorphous silica content (above 99%) not only enhance concrete strength and durability but also reduce cement demand, offering a sustainable pathway to lower CO_2_ emissions in construction^18^.

Recent breakthroughs in nanoscience and nanomaterials have revolutionized the synthesis of high-purity MS from RHA. Advanced processing techniques now enable precise control of particle size (20–50 nm) and amorphous silica content (> 99%), unlocking enhanced pozzolanic reactivity for next-generation cement composites^19^. Compared to conventional cementitious materials, MS (amorphous) significantly enhances concrete performance through its fine particle size and high reactivity, improving strength, reducing permeability, and increasing durability via pozzolanic activity and pore refinement^20^. Moreover, using extracted micro silica (EMS) from agricultural waste (RHA) as a partial cement replacement at various percentages enhanced compressive strength and mitigated alkali-silica reactivity^18^. Previous studies indicate that incorporating EMS at low replacement levels (5–15%) consistently enhances compressive strength across all curing periods when compared to traditional mixtures^18,21,22^. Research indicates that RHA’s amorphous silica densifies concrete by refining the interfacial transition zone, with 10–20% replacement, optimizing strength and durability^23^. Research shows that RHA effectively enhances cement properties when added at up to 30%, improving workability, optimizing setting time, and reducing permeability due to its pozzolanic reactivity^24^. High cement substitution with RHA has led to reduced mechanical strength, which can be addressed by developing ternary blends incorporating other high-reactivity SCMs^25,26^. Ternary concrete, which utilizes a combination of supplementary materials, enhances mechanical strength and promotes sustainability by reducing CO_2_ emissions through partial replacement of traditional cement^27,28^. Studies recommend combining RHA with higher-reactivity SCMs, such as MS, to optimize mix designs where RHA exceeds 20% cement replacement, improving both pozzolanic reactivity and particle packing density^29^. Research shows that geopolymers with 15% or more RHA develop stronger compressive strength than fly-ash blends, indicating that RHA’s silica content is more effective for high-performance applications^30^. Therefore, to overcome the strength decline issues associated with high cement replacement, this study pioneers a novel ternary system incorporating RHA and EMS. The study investigates the strength-performance trade-off in high-volume SCM mixtures, providing a pathway for durable, low-carbon concrete.

However, establishing optimal dosage parameters remains problematic when studies examine limited size ranges and concentration levels. Furthermore, the conventional experimental design method often requires a large number of tests, which can be both expensive and time-consuming, to evaluate how different variables at various levels influence concrete properties^31^. RSM offers an efficient statistical approach for predictive modeling, enabling a comprehensive assessment of factor effects on target responses with minimal experimental runs. This design-of-experiments technique significantly reduces resource requirements while maintaining robust evaluation of variable interactions^32,33^. The statistical validity of RSM models is evaluated through multiple metrics, including the coefficient of determination (R²), adjusted R², predicted R², p-values, adequate precision ratios, and residual diagnostics^34,35^. Researchers have successfully employed RSM to design experiments, predict performance, and optimize concrete mixtures containing SCMs. These studies consistently demonstrate high predictive accuracy for key concrete properties, confirming RSM’s reliability in cementitious system analysis^36–39^. The reliability of RSM extends to nanomaterial-modified concrete, as demonstrated by Adamu et al^40^, who reported statistically significant models for graphene-enhanced fly ash concrete. Optimization-driven studies, such as the development of geopolymer-based artificial angular aggregates using cut-blade techniques, highlight the growing use of statistical and computational tools to enhance sustainable concrete materials^41^. Mohammed et al^42^, employed RSM to analyze the effects of nano-silica (NS) in pervious geopolymer concrete. Similarly, Hamada et al^43^, utilized palm oil fuel ash nanoparticles to optimize lightweight concrete systems. The significance of creating robust and optimal cementitious systems utilizing cutting-edge computational methods is highlighted by recent developments in concrete behavior modeling and prediction analysis^44–46^.

Artificial Neural Networks have emerged as a powerful supervised machine learning tool for predictive modeling and optimization in concrete technology^1,47^. As robust computational systems capable of identifying complex nonlinear patterns, ANNs excel at optimizing concrete mix designs by analyzing relationships between key parameters. These models have demonstrated particular effectiveness in determining optimal proportions of aggregates, water-cement ratios, and SCMs to achieve targeted performance characteristics^48^. ANNs are biologically inspired machine learning models composed of interconnected computational neurons that learn complex nonlinear relationships through iterative weight adjustments during training, enabling robust pattern recognition and predictive capabilities across engineering domains^49^. The study by Oyebisi et al^50^, on the evaluation of ternary blended concrete strength demonstrates the effective use of artificial intelligence techniques to predict and optimize concrete performance. Advanced machine-learning approaches are increasingly being applied in sustainable concrete research, as shown by studies predicting the durability of geopolymer concrete with CDW and artificial aggregates under aggressive exposure, highlighting the expanding role of ANN-based modelling^51^. Chithra et al^52^, successfully modeled the strength properties of ultra-high-performance concrete, while Duan et al^53^, applied ANNs to predict the compressive strength of recycled aggregate concrete. The increasing significance of computational methods for precisely predicting material behavior and optimizing mix design is further highlighted by recent developments in AI-driven concrete modeling, such as deep learning-based damage prediction under compression^54^. Microstructural characterization and data-driven modeling are increasingly being combined in emerging research to better understand the performance patterns of cementitious materials^55–57^. These advancements further support the study’s use of combined RSM and ANN frameworks, which are crucial for accurately optimizing ternary concrete performance and capturing complex material interactions.

Although significant research has explored the individual use of RHA and EMS in concrete, a clear knowledge gap exists regarding their synergistic effects, particularly at high-volume replacement levels (> 30%). Furthermore, the optimization of such ternary blends has been limited by the lack of advanced predictive modelling. Specifically, applying proven techniques such as RSM and ANN to high-dosage RHA-EMS mixtures represents a critical, unexplored area of research. Ultimately, this scarcity of optimized models and a fundamental understanding of the EMS-RHA microstructural interactions impairs the development of practical, sustainable, and high-performance concrete formulations.

To address these gaps, this work comprehensively investigates the mechanical, microstructural, and sustainability performance of EMS–RHA ternary concrete at higher replacement levels. Using predictive modeling frameworks for optimization, the research investigates various concrete formulations, including a control mix (CM) and twelve ternary mixtures incorporating RHA and EMS. The compressive strength was evaluated with varying percentages of EMS (5%, 10%, and 15%) and RHA (5–40%) at different replacement levels. SEM analysis was performed to examine microstructural features, including pore structure and particle agglomeration. Additionally, RSM and ANN models were employed to identify optimal EMS and RHA dosages, enabling precise determination of the optimized mix proportions to achieve maximum compressive strength, material efficiency, and sustainability. Their implementation directly reduces cement consumption and valorizes agro-waste, thereby bridging the critical gap between advanced material science and sustainable construction.

## Materials and methods

**Fig. 1:**
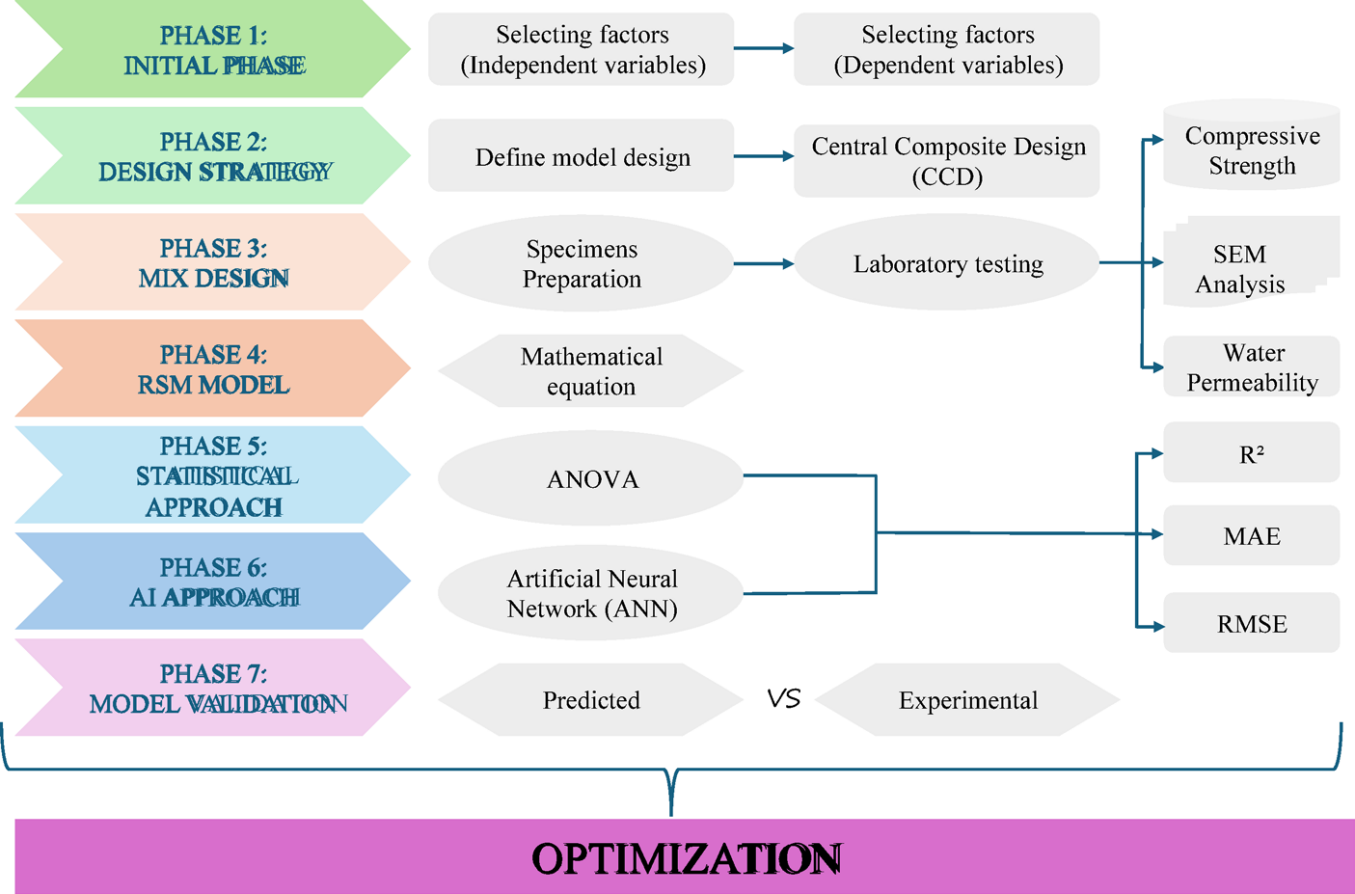
Schematic overview of the experimental and modelling workflow used for EMS–RHA concrete optimization, combining CCD-based design, RSM, ANN, and validation steps.

This study employs a systematic approach, as illustrated in Fig. 1, to analyze and optimize the effect of EMS and RHA on the compressive strength of cementitious concrete composites. Concrete samples were prepared with varying EMS dosages (5%, 10%, and 15%) using different percentages of RHA, varying from 5% to 40%. Their compressive strength was assessed at 14, 28, and 56 days. SEM scans were used to investigate the morphology and agglomerates resulting from the high cement replacement. The experimental data were analyzed using RSM and an ANN to predict compressive strength. RSM is a statistical and mathematical modeling technique commonly employed for experimental design and optimization in engineering applications^58^. The ANN model was optimized using hyperparameters determined through iterative testing. Model predictions were evaluated using various statistical metrics. The trained networks were then used to determine optimal EMS and RHA dosages to maximize compressive strength.

### Materials

The primary materials used in this study were ordinary Portland cement (OPC), conforming to ASTM standards and sourced from Bestway Cement Factory, Pakistan, along with EMS and RHA obtained from Charsadda District, Khyber Pakhtunkhwa, Pakistan. Rice husk (RH) was collected from Charsadda, selected based on its climate and geographic conditions. The RH was incinerated at 600–700 °C in a ferrocement drum for 24 h to achieve high silica content, producing approximately 22% rice husk ash (RHA). After cooling, the ash was ground in a rotary mill for 12 h at 15 rpm and stored in polythene bags for microstructural investigations. The RHA sample was subsequently processed into micro‑silica (EMS) using an optimized, cost‑effective method at the PCSIR laboratory^18^. Table 1 lists the physical composition of OPC, EMS, and RHA.

**Table 1:** Physical characteristics of OPC, EMS, and RHA used in the mix formulations.

	Specific gravity (Kg/m^3^)	Particle size (nm)	SiO_2_ (%)
OPC	3.15	-	21.00
EMS	2.15	487.5	99.470
RHA	2.83	1273	91.028

**Fig. 2:**
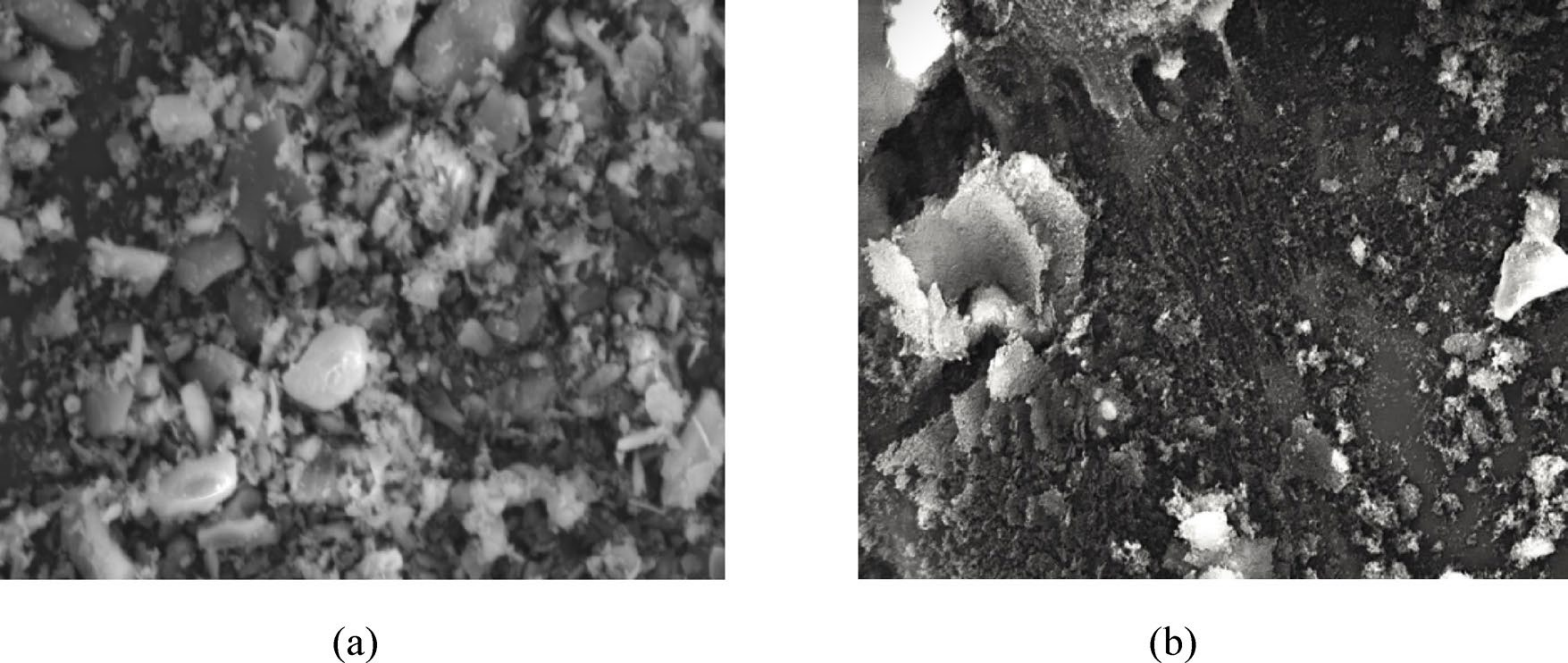
SEM micrographs of **(a)** RHA and **(b)** EMS, illustrating differences in particle morphology.

Figure 2 presents SEM micrographs obtained using a Nova 450 Nano-SEM, illustrating the morphological characteristics (particle shape and size) of RHA and EMS samples. The EMS produced through chemical extraction at PCSIR exhibits a very high silica purity, with a SiO_2_ content of 99.47%, confirming the effectiveness of the extraction process. The chemical compositions of the raw materials are summarized in Table 2. The SCMs (EMS and RHA) are characterized by exceptionally high silica (SiO_2_) content of 99.47% and 91.02%, respectively. In contrast, the OPC has a high calcium oxide (CaO) content of 61.70%, consistent with conventional cement chemistry. In accordance with ASTM C618, both EMS and RHA satisfy the chemical requirements for Class N pozzolans. The combined contents of SiO_2_, Al_2_O_3_, and Fe_2_O_3_ in EMS and RHA are 99.53% and 93.16%, respectively, which significantly exceed the minimum 70% threshold specified by the standard. As a result, these materials exhibit strong pozzolanic reactivity during cement hydration, forming additional calcium-silicate-hydrate (C-S-H) gel. This secondary C-S-H formation leads to microstructural densification, which underpins the observed enhancements in mechanical strength and durability of the concrete mixtures. Moreover, the substantially finer particle size of EMS (487.5 nm) compared to RHA (1273 nm) provides more nucleation sites, accelerating hydration reactions and promoting the development of a more refined and homogeneous microstructure. The combined chemical reactivity and physical filler effects of EMS and RHA therefore contribute synergistically to improved mechanical performance and long-term durability, confirming their effectiveness as sustainable supplementary cementitious materials in ternary concrete systems.

**Table 2:** Chemical oxide composition (% by weight) of RHA, EMS, and OPC.

Oxide composition (%)	RHA	EMS	OPC
SiO_2_	91.02	99.47	21.00
CaO	2.22	0.25	61.70
Al_2_O_3_	ND	ND	5.04
Fe_2_O_3_	2.13	0.06	3.24
P_2_O_5_	1.04	ND	ND
MgO	ND	ND	2.56
SO_3_	0.47	0.11	1.51
K_2_O	2.78	0.05	0.62
Na_2_O	ND	ND	0.13
TiO_2_	0.06	0.007	ND
MnO	0.13	0.006	ND
CuO	0.01	0.008	ND
LOI	ND	ND	1.83
SiO_2_ **+** Al_2_O_3_ **+** Fe_2_O_3_	93.16	99.53	29.28
ASTM C618	Class N	Class N	NA

Note: ND = Not detected (below XRF detection limit); NA = Not applicable. The sum of SiO_2_, Al_2_O_3_, and Fe_2_O_3_ is used for pozzolanic classification in accordance with ASTM C618.

### Methods

#### Mix proportioning and testing

This study involved preparing 13 concrete mixtures with varying percentages of EMS and RHA. EMS were utilized at three different dosages (low (5%), medium (10%), and high (15%)) to assess their influence on high percentage replacement by RHA, as shown in Table 3.

**Table 3:** EMS and RHA replacement levels are grouped into low (LD), medium (MD), and high (HD) dosages.

Dosages	Dosage replacement by mass of OPC (%)				
	EMS	RHA			
Low Dosage (LD)	5%	5%	10%	15%	20%
Medium Dosage (MD)	10%	15%	20%	25%	30%
High Dosage (HD)	15%	25%	30%	35%	40%

The dosages of EMS and RHA were selected based on their pozzolanic reactivity and their impact on the mechanical performance of the concrete mixtures. The EMS dosage (5–15%) was chosen to optimize compressive strength, as EMS exhibits high reactivity and fine particle size, enhancing pozzolanic activity without adversely affecting workability^18,21,22^. Similarly, the RHA replacement levels (15%−35%) were selected, based on previous studies showing their effectiveness in improving strength, durability, and permeability at these levels, while minimizing negative effects on workability and setting time^59^. The dosages were adjusted to ensure the maximum cement replacement without compromising the mechanical performance of the concrete mixtures. Both EMS and RHA were used as partial cement replacements on a mass basis, in line with standard practices in SCM research. This approach ensures consistent binder proportions, making it easier to compare with previous studies. Volumetric substitution was avoided because density variations between OPC and SCMs could introduce variability that is inconsistent with cement industry practices. The mix proportions for the concrete mixtures were designed following the ACI 211.1^60^ standard for concrete mix design, which is widely used for proportioning materials to achieve target strength and workability. To maintain consistent workability across all concrete mixtures, a superplasticizer was used, maintaining a slump of 120 ± 30 mm. This adjustment allowed us to focus on the effects of varying EMS and RHA dosages on compressive strength while maintaining constant workability. The detailed proportions of EMS and RHA replacements are given in Table 4. The compressive strength of concrete samples was evaluated in accordance with ASTM C192^61^. Cylindrical specimens (100 mm diameter × 200 mm height) were cast using specialized steel moulds in accordance with ASTM C39^62^. After demolding at 24 h, the samples were moist-cured in lime-saturated water at 20 °C until testing at 14, 28, and 56 days. For each mixture, three identical specimens were subjected to uniaxial compression testing in accordance with ASTM protocols, with the mean compressive strength values reported.

**Table 4:** Mix proportions and fresh properties of the ternary concrete mixtures.

Mix ID	W (Kg/m^3^)	Binder, b (Kg/m^3^)			Aggregates, (Kg/m^3^)			Superplasticizer (% of b)	Slump (mm)
		OPC	EMS	RHA	FA	CA 20 mm	CA 10 mm		
CM (M1)	157.5	450.0	-	-	700	682	418	1.0	120 ± 30
EMS (05%) and RHA (05%) (E05R05) M2		400.0	22.5	22.5				1.2	
EMS (05%) and RHA (10%) (E05R10) M3		382.5	22.5	45.0				1.2	
EMS (05%) and RHA (15%)(E05R15) M4		360.0	22.5	67.5				1.2	
EMS (05%) and RHA (20%)(E05R20) M5		337.5	22.5	90.0				1.2	
EMS (10%) and RHA (15%)(E10R15) M6		337.5	45.0	67.5				1.3	
EMS (10%) and RHA (20%)(E10R20) M7		315.0	45.0	90.0				1.3	
EMS (10%) and RHA (25%)(E10R25) M8		292.5	45.0	112.5				1.3	
EMS (10%) and RHA (30%)(E10R30) M9		270.0	45.0	135.0				1.3	
EMS (15%) and RHA (25%) (E15R25) M10		270.0	67.5	112.5				1.4	
EMS (15%) and RHA (30%)(E15R30) M11		247.5	67.5	135.0				1.4	
EMS (15%) and RHA (35%)(E15R35) M12		225.0	67.5	157.5				1.4	
EMS (15%) and RHA (40%)(E15R40) M13		202.5	67.5	180.0				1.4	

#### Scanning electron microscopy

The microstructural characteristics of cement pastes were analyzed using SEM(JSM-IT100, NCEG, University of Peshawar). To observe SEM, slices of hardened paste were prepared and dried using a solvent-exchange method with isopropanol, then sputter-coated with a thin layer of gold to ensure adequate conductivity. A scanning electron microscope was used, in which the secondary electron mode was used with an accelerating voltage of 20 kV and a working distance of 10 mm, with a magnification ranging between 500x and 10,000x, to obtain high-resolution imaging.

#### Concrete permeability

**Fig. 3:**
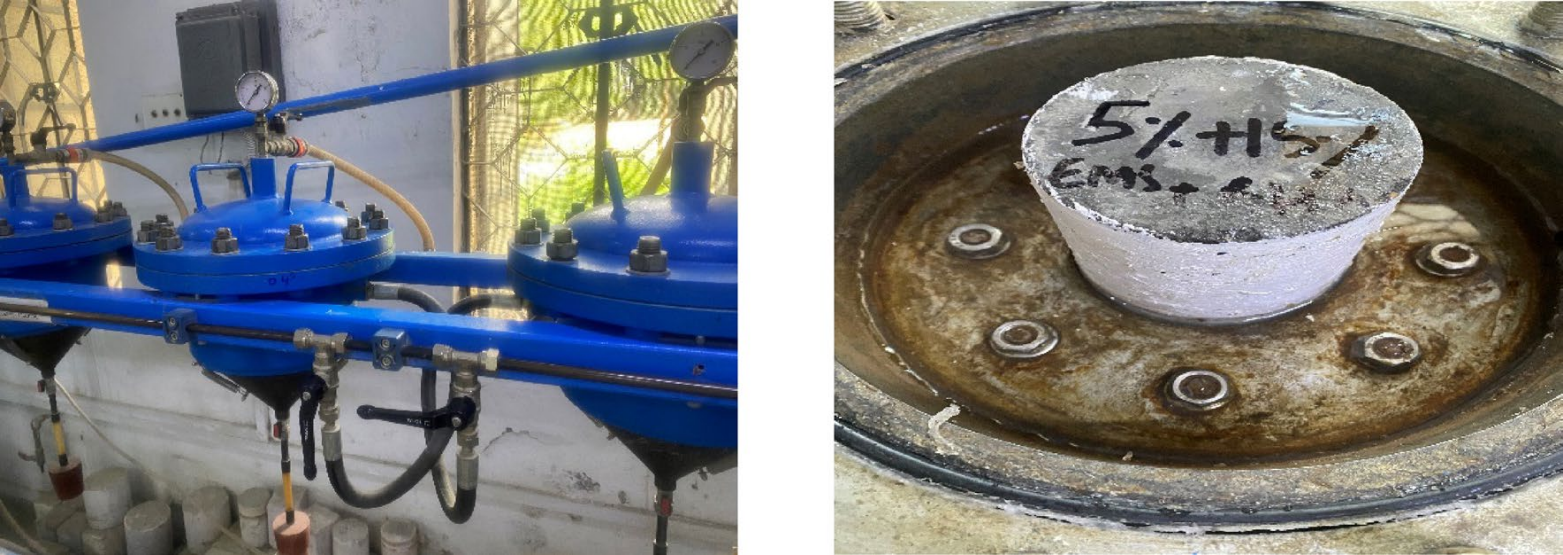
Test setup for determining the water-permeability coefficient.

In addition to the compressive strength, water permeability tests were conducted for four concrete mixtures highlighted in the SEM analysis: CM, E05R15, E10R25, and E15R35. A water penetration test was performed at the University of Engineering and Technology, Taxila, Pakistan. Three samples from each mixture were tested to determine the water penetration depth in accordance with DIN 1048^63^. For this test, concrete cylindrical samples with a 100 mm (diameter) and 150 mm (height) were water cured for 56 days of age. The apparatus consists of a metallic frame with four cells designed to hold specimens intended for testing, as shown in Fig. 3. Each cell is equipped with a monometer that regulates internal pressure. A rechargeable compensation chamber ensures consistent pressure throughout all testing processes. The pressure can be adjusted from 0 to 30 bars using an automatic pump with variable output, providing optimal conditions for the specimen under evaluation. The samples were subjected to hydrostatic stress for the duration of the present period (72 h). In unsaturated media, such as partially wet concrete, the penetration of water under external hydraulic pressure is correlated to unsteady flow and sorptivity^64,65^. Thus, this methodology measures transportation driven by a pressure gradient and can be used to evaluate permeability under transient flow conditions^65^. The test assumes that water penetration does not begin until a stable flow condition has been established^66,67^. The applied pressure and measurement time were sufficient to yield results similar to those under fully saturated conditions, even in low-permeability concrete. Uniaxial penetration is also supported by the specimens’ high density and the low penetration depth relative to the test area diameter. As a result, this method allows for the determination of the coefficient of permeability. Based on the Darcy law, the penetration coefficient can be calculated according to Eq. (1) given below:

1 $$k\,=\,Qh/AtP$$

while ‘*Q’* is the simple discharge, ‘*P’* the hydrostatic pressure, ‘*A*’ the top surface area, ‘h’ the sample’s height, and ‘*t*’ the water permeability time.

#### Response surface methodology

In this study, the Central Composite Design (CCD) approach within Response Surface Methodology (RSM) was used to optimize the dosages of EMS and RHA in ternary concrete mixtures. CCD in Design-Expert (Version 12) was selected for its ability to efficiently capture quadratic relationships among multiple factors and identify optimal mix compositions while minimizing the number of experimental trials. This technique enables robust modeling of interactions between EMS and RHA dosages, enabling accurate predictions of compressive strength in ternary blends. This approach utilizes designed experiments to establish predictive models for parameter optimization. A series of experiments was conducted to examine the relationships between input (factor) variables and target response variables. RSM is an efficient statistical approach for experimental design, analysis, optimization, and validation. In concrete research, RSM has been extensively applied to experimental design, statistical modeling, and the evaluation of factor-response relationships. The initial phase of RSM involves designing an experimental matrix that can characterize the response surface. In this study, the independent variables comprised different types of SCMs (EMS and RHA) and their respective dosages, while the dependent variables were defined as compressive strength measurements at 14, 28, and 56 days of curing. The RSM framework was implemented to evaluate age-dependent mechanical performance in cementitious concrete composites through compressive strength analysis. During model development, the system autonomously selected optimal regression terms (linear or higher-order polynomials) based on detected parameter correlations, thereby generating statistically significant predictive relationships for composite strength properties. RSM operates by modeling parameter relationships using higher-order polynomial equations, as shown in Eq. (2).

2 $${\mathrm{Y}}\,{\mathrm{=}}\,C\,{\mathrm{+}}\,{{\mathrm{A}}_1}\left( {{{\mathrm{X}}_1}} \right)\,+\,{{\mathrm{A}}_2}\left( {{{\mathrm{X}}_2}} \right)\,+\,{{\mathrm{A}}_3}\left( {{{\mathrm{X}}_1}{{\mathrm{1}}^2}} \right)\,+\,{{\mathrm{A}}_4}\left( {{{\mathrm{X}}_{\mathrm{2}}}{{\mathrm{1}}^{\mathrm{2}}}} \right)\,+\,{{\mathrm{A}}_5}\left( {{{\mathrm{X}}_1}^*{\text{ }}{{\mathrm{X}}_2}} \right)$$

The RSM model (Eq. 2) relates compressive strength (Y) at 14–56 days to EMS (X_1_) and RHA (X_2_), with coefficients A_1_-A_5_ and intercept C. A 33-run experimental design with five center point replicates was implemented. Concrete composites were prepared according to this matrix, and compressive strength data were analyzed using Design-Expert software. The concrete composites were systematically analyzed and optimized using statistical modeling. Model performance for compressive strength was evaluated using ANOVA, with p-values, F-values, and lack-of-fit errors assessed to validate the adequacy of the regression model. Design-Expert generated both 2D and 3D response surface plots to visualize the interactive effects of the two key factors. Table 5 presents the statistical data for the independent variables (EMS and RHA) and the dependent variable (compressive strength). The data includes the range, mean, and standard deviation for each variable, providing insights into the data’s variability and distribution used for optimization. Statistical analysis highlights significant variability in the independent variables (EMS and RHA) and their effects on compressive strength, underscoring the robustness of the optimization process and the potential to improve concrete performance through precise dosage adjustments.

**Table 5:** Statistical summary of the input variables and output response for the RSM Model.

Variable Type	Variable Name	Unit	Minimum	Maximum	Mean	Standard Deviation
Independent	RHA	Kg/m^3^	0.0	184.0	80.6	53.9
Independent	EMS	Kg/m^3^	0.0	67.5	33.8	24.5
Dependent	CS	MPa	18.65	44.05	30.18	6.67

#### ANN modelling

ANNs were developed on the principle that biological neural processing fundamentally differs from conventional computing. These networks consist of interconnected, parallel-processing units organized into input, hidden, and output layers. The architecture requires matching input/output neurons to corresponding parameters and target variables, while hidden layer neurons are optimized through systematic iteration to achieve peak performance^68^. In MATLAB, the ANN model is trained by supplying it with input and output data. The model adjusts its weights and biases to minimize the error by comparing the predicted output with the desired output^69^. The dataset was randomly split into 70% training and 30% validation sets. Various configurations of hidden neurons were systematically evaluated to determine the best hyperparameters for the ANN model^70^. The Levenberg-Marquardt algorithm was employed to optimize the model’s weights and biases, as it is an efficient backpropagation method widely recommended for supervised learning tasks^71^. The network architecture employed tan-sigmoid activation functions in the hidden layer (optimized to 9 neurons) and linear functions in the output layer. Comparative analysis revealed that the tan-sigmoid function outperformed log-sigmoid alternatives in hidden-layer applications, consistent with the established literature. Model performance was quantitatively assessed using three key metrics: root mean square error (RMSE), mean absolute error (MAE), and correlation coefficient (R), providing a comprehensive evaluation of prediction accuracy and model reliability^72^. The ANN model was selected for its proven ability to capture nonlinear and multivariate relationships that are difficult to model with conventional regression models. The combined synergistic effect of EMS and RHA on compressive strength arises from complex interactions involving pozzolanic activity, filler effects, particle fineness, and age-dependent hydration kinetics. Such nonlinear behaviour makes ANN an appropriate choice, as its adaptable architecture enables the network to learn intricate patterns from experimental data without requiring explicit assumptions about variable relationships. Compared to other machine-learning algorithms, such as decision trees or support vector regressors, ANNs offer greater flexibility in modelling continuous material properties and have demonstrated superior performance in predicting cementitious system performance in recent literature^73–75^. Furthermore, using a single hidden layer with an optimized neuron count balances predictive accuracy and generalization, preventing overfitting while maintaining computational efficiency^76^. These include the R, MAE, and RMSE. These indicators are widely used within the field to quantify predictive accuracy and error magnitude^77^. Lower values of MAE and RMSE signify better model performance, reflecting higher predictive efficiency and reduced deviation from observed values^78^.

#### Performance metrics

The proposed model was evaluated using statistical parameters such as the R, R², RMSE, MAE, and relative absolute error (RAE). These are defined by the following Eqs. (3–7).

3 $$\:R\:=\frac{{\sum\:}_{i=1}^{n}\:\left({y}_{i}-\stackrel{-}{y}\right)\left({y}_{i}^{{\prime\:}}-\stackrel{-}{y}{\prime\:}\right)\:}{\sqrt{{\sum\:}_{i=1}^{n}{\left({y}_{i}-\stackrel{-}{y}\right)}^{2}{\sum\:}_{i=1}^{n}{\left({y}_{i}^{{\prime\:}}-\stackrel{-}{y}{\prime\:}\right)}^{2}}}$$

4 $$\:{\mathrm{R}}^{2}=\:1-\frac{{{\sum\:}_{\mathrm{i}=1}^{\mathrm{n}}\left({\mathrm{y}}_{\mathrm{i}}^{{\prime\:}}-{\mathrm{y}}_{\mathrm{i}}\right)}^{2}\:\:}{{\sum\:}_{\mathrm{i}=1}^{\mathrm{n}}{\left({\mathrm{y}}_{\mathrm{i}}-\stackrel{-}{\mathrm{y}}\right)}^{2}}$$

5 $$\:RMSE=\:\sqrt{\frac{{\sum\:}_{i=1}^{n}\:{\left({y}_{i}^{{\prime\:}}-{y}_{i}\right)}^{2}}{n}}$$

6 $$\:MAE=\frac{1}{n}\:{\sum\:}_{i=1}^{n}\:|{y}_{i}^{{\prime\:}}-{y}_{i}|\:$$

7 $$\:RAE\:=\frac{{\sum\:}_{i=1}^{n}|{y}_{i}^{{\prime\:}}-{y}_{i}|}{{\sum\:}_{i=1}^{n}|{y}_{i}-\stackrel{-}{y}|}\:$$

where: $\:{y}_{i}$ represents actual observed values, $\:{y}_{i}^{{\prime\:}}$ represents the predicted values, $\:\stackrel{-}{{y}^{{\prime\:}}}$ is the mean of predicted values, $\:\stackrel{-}{y}$ is the mean of the actual values, and $\:n$ is the total number of data points.

## Results and discussion

### Influence of EMS and RHA on the compressive strength with aging

**Fig. 4:**
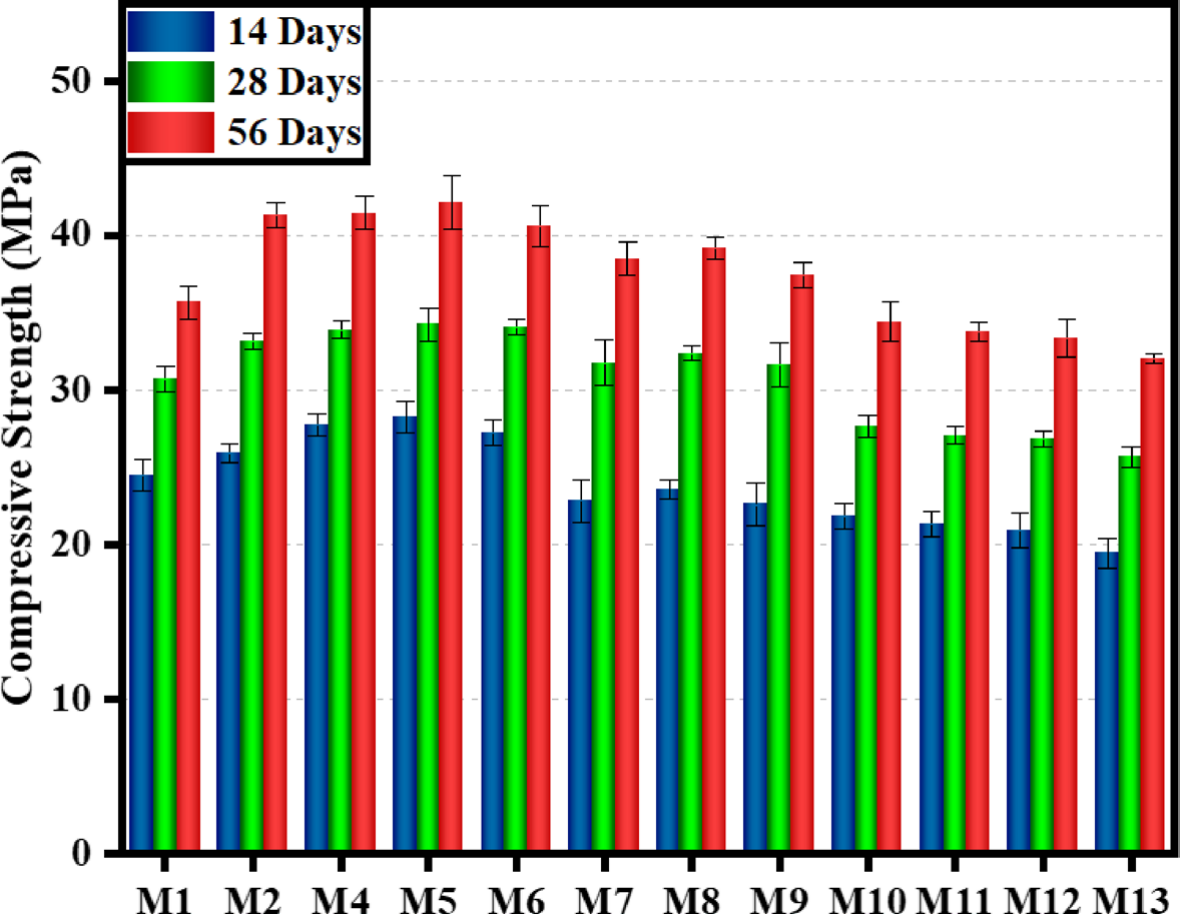
Comparison of compressive strength results of CM and ternary-based mixes.

The study examines the impact of EMS and RHA dosages on the age-dependent compressive strength of cement concrete composites at three dosage levels (low, medium, and high) during 14-, 28-, and 56-day curing periods. To increase sustainability, a large-scale substitution of cement with RHA was implemented, and various levels of EMS were added to compensate for the corresponding loss of strength. Each mixture was tested with at least 3 replicates at 14, 28, and 56 days, and results are presented as mean ± standard deviation. Error bars representing ± 1 SD are included in all strength plots to reflect variability and statistical reliability. Since workability was kept constant at 120 ± 30 mm, no variations in slump values were observed across the mixtures. The use of superplasticizer ensured that the mixtures’ fresh properties remained consistent, enabling a focused comparison of compressive strength development. This research evaluates the mechanical performance of ternary-blended mixtures compared to conventional control mixes, aiming to determine the optimal cement replacement ratio that maintains or enhances structural strength. Figure 4 displays the variation in compressive strength between results for CM and ternary-based samples with different percentages of EMS and RHA (CM (M1), E05R05 (M2), E05R10 (M3), E05R15 (M4), E05R20 (M5), E10R15 (M6), E10R20 (M7), E10R25 (M8), E10R30 (M9), E15R25 (M10), E15R30 (M11), E15R35 (M12), and E15R40 (M13)). The main aim of the investigation is to establish a high-performance, sustainable concrete formulation by carefully assessing whether ternary mixtures can approach, or even outperform, the strength of the control mix, thereby achieving the maximum achievable cement replacement threshold. Before testing, the samples were cured at 20 °C until the designated testing age was reached. The results reveal a clear performance trend across curing ages. A comparative analysis of compressive strength development between the CM and ternary blended mixtures at 14, 28, and 56 days of curing. Test results showed that mixes with 5–10% EMS and moderate RHA dosages achieved higher compressive strength than the CM across all curing ages. Incorporation of EMS and RHA at low-to-moderate replacement levels significantly enhanced strength, with the optimum observed for the 5% EMS + 15% RHA blend (E05R15, M4), which achieved 42.08 MPa at 56 days, representing an 18% improvement compared to the control. Similar enhancements were observed in E05R10 (M3) and E05R20 (M5), confirming the beneficial synergy between EMS and RHA at these dosages. At higher dosages, particularly in the 10% EMS + 25% RHA blend (E10R25, M8), strength gains were still above the CM, though slightly reduced relative to the optimum. Conversely, mixes with 15% EMS and ≥ 25% RHA (M10-M13) demonstrated lower compressive strengths than the control across all ages. This decline is attributed to binder dilution, excessive silica, particle agglomeration, poor dispersion, and increased porosity. Conversely, EMS with a high dosage of 15% combined with 35% RHA achieved reduced strength (19.42 MPa, 25.62 MPa, and 31.99 MPa at the same intervals). However, exceeding 30% combined replacement (EMS + RHA) reduced strength performance. The substantial strength enhancement observed at low and medium EMS and RHA dosages primarily stems from the formation of a more compact Calcium Silicate Hydrate (C-S-H) gel matrix, as confirmed by SEM analysis. This finding aligns with existing research demonstrating that SCMs containing highly reactive amorphous silica, particularly those with optimized particle fineness and pozzolanic activity, can substantially improve the mechanical properties of cementitious composites^79,80^. The observed strength reduction resulted from retarded pozzolanic reactions that disrupted normal hydration kinetics, creating a more porous microstructure that compromised mechanical performance^81^. The observed decrease in compressive strength at elevated dosage levels primarily results from insufficient dispersion of EMS and RHA particles, leading to pronounced agglomeration. This trend of diminishing mechanical performance correlated strongly with increasing EMS and RHA dosages. The high cement replacement ratio reduces compressive strength due to excessive silica, leading to particle agglomeration that hinders hydration and weakens the matrix. Notably, E15R40 mixtures exhibited the lowest compressive strength among CM and other ternary-based mixtures. This decrease in mechanical performance can be attributed to particle agglomeration phenomena. The ultrafine particles of EMS promote agglomeration, creating localized void structures and consequently reducing the load-bearing capacity^82^. The high fineness and reactive amorphous silica content of SF are widely used for enhancing concrete strength through additional pozzolanic reactions^80^. The same was reported by Lima^21^ and Cavalcante^22^, who observed higher compressive strength in cement pastes containing 5% and 16% RHA-derived silica, respectively. The early-age improvement can be attributed to improved hydration and efficient void filling by the EMS, whereas the long-term strengthening can be attributed to the pozzolanic activity of RHA. The strength enhancement observed in EMS–RHA ternary blends can be attributed to complementary mechanisms, including the early filler–nucleation effect of EMS and the long-term pozzolanic reactivity of RHA. This behaviour is consistent with findings from other ternary SCM systems such as RHA–GGBFS and RHA-Metakaolin, where synergistic interaction between a highly reactive amorphous alumino-silicate (e.g., metakaolin or slag) and RHA improves both early and later-age strength. In RHA–GGBFS systems, the latent hydraulic activity of slag accelerates secondary C–S–H formation, while RHA contributes additional siliceous gel, producing a refined pore structure^7^. Similarly, RHA–Metakaolin blends benefit from metakaolin’s high alumina content, forming C–A–S–H gel phases that enhance matrix densification^83,84^. Compared to these systems, the EMS–RHA combination follows a similar trend, with EMS acting as a microfiller and reactive silica source at early ages, while RHA contributes to long-term strength through continued pozzolanic activity. Thus, the superior strength observed at 56 days in optimized EMS–RHA mixtures aligns with the well-established behaviour of highly reactive SCM-based ternary binders, validating the mechanistic explanation of the synergistic improvement.

The E10R25 combination is the most suitable blend where durability is the key factor. The practical feasibility is established by a strength gain of 5.98%, which meets code requirements, and by its exceptional strength, evident in the low porosity and a more compact C-S-H matrix than in the standard mix. This is a specifically designed formulation that uses agro-waste to the fullest without compromising workability and is intended for applications such as marine exposures, where durability takes precedence over small incremental gains in strength.

Additionally, a one-way ANOVA was performed to investigate the impact of the mix designs on compressive strength. The compressive strength of concrete was significantly influenced by mix design at all testing ages, as confirmed by a one-way ANOVA (14-day: F(12, 26) = 29.76, *p* < 0.001; 28-day: F(12, 26) = 26.70, *p* < 0.001; 56-day: F(12, 26) = 31.58, *p* < 0.001) as presented in Table 6.

**Table 6:** One-way analysis of variance (ANOVA) for the effect of mix design on compressive strength.

Days	Source of Variation	SS	df	MS	F	*P*-value
**14**	Between Groups	282.0292	12	23.50244	29.75806	4.01E-12
	Within Groups	20.53438	26	0.789784		
**28**	Between Groups	243.8999	12	20.32499	26.70216	1.43E-11
	Within Groups	19.79053	26	0.761174		
**56**	Between Groups	418.1419	12	34.84516	31.57776	1.99E-12
	Within Groups	28.69026	26	1.103471		

Note: (SS) Sum of Squares, (df) degree of freedom, (MS) Mean Squares. All ANOVA models were statistically significant at *p* < 0.001.

The post-hoc Tukey HSD analysis was conducted across all testing ages; however, only the 28-day results are reported, as the 28-day compressive strength is the standard benchmark age for evaluating concrete performance and comparing the effectiveness of mix designs. The 28-day strength Tukey test revealed five distinct statistical groupings (A-E), establishing a clear performance hierarchy (Table 7). The results revealed that moderate additive combinations (Group A) yielded the highest compressive strength, forming a statistically superior group. The control mix (CM, Group C) significantly outperformed by Group A but was itself significantly stronger than all high-additive mixes (Groups D and E). This confirms an optimal dosage range for the additives, beyond which (≥ 15%) a significant detrimental effect on structural performance occurs.

**Table 7:** Statistical grouping of concrete mixes based on Tukey HSD post-hoc test for 28-day compressive strength.

Mix IDs	Statistical Group
**E05R05 (M2)**,** E05R10 (M3)**,** E05R15 (M4)**,** E05R20 (M5)**	A
**E10R15 (M6)**,** E10R20 (M7)**,** E10R25 (M8)**,** E10R30 (M9)**	B
**CM (M1)**	C
**E15R25 (M10)**,** E15R30 (M11)**,** E15R35 (M12)**,	D
**E15R40 (M13)**	E

Note: Mixes sharing the same letter are not statistically different (*p* > 0.05), according to the Tukey test.

### SEM analysis

**Fig. 5:**
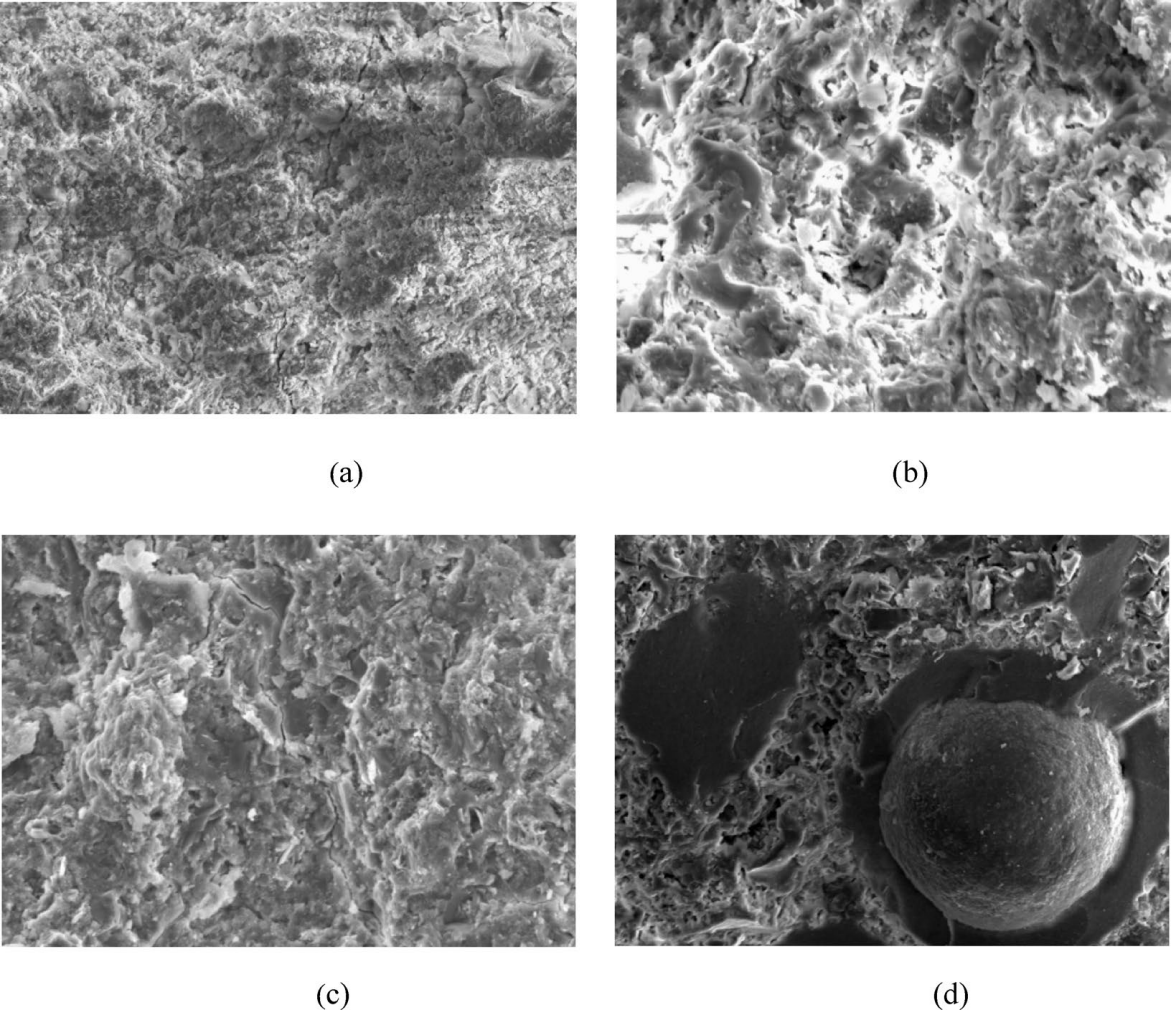
SEM microstructures of: **(a)** control mix (CM), **(b)** E05R15, **(c)** E10R25, and **(d)** E15R35, highlighting progressive microstructural densification with increasing EMS and RHA contents.

Microstructural characterization of cement pastes was conducted using a JSM-IT100 scanning electron microscope at the National Centre of Excellence in Geology (NCEG), University of Peshawar. The SEM micrographs reveal distinct morphological differences between the control paste and the ternary blended mixtures, as shown in Fig. 5. Microstructural analysis reveals that specimens containing 5% EMS with 15% RHA (E05R15) and 10% EMS with 25% RHA (E10R25) developed significantly denser matrices compared to conventional cement paste. This enhanced compaction likely stems from EMS’s pronounced pozzolanic reactivity, which promotes the formation of high-density hydration products and microstructural refinement. However, the high replacement ratio (E15R35) exhibited three critical microstructural defects under SEM examination: cement hydration deficiencies, MS agglomerates, and void networks. These phenomena collectively indicate, exceeding the optimal binder replacement threshold and dispersion limitations ultimately leads to a porous, low-density matrix. Excessive silica content hinders cement hydration, leading to particle agglomeration, unreacted cement, and increased porosity, ultimately reducing strength and durability. Excessive reactive silica content can disrupt cement hydration kinetics, leading to incomplete consumption of anhydrous cement. This incomplete hydration phenomenon promotes MS agglomeration and the formation of large voids. As a result, ultimately compromising the material’s mechanical strength and long-term durability^85^. Microstructural analysis at varying magnifications reveals distinct differences between agglomerated regions and the surrounding hydrated cement matrix. The clustering of MS particles reduces the effective reactive surface area, leading to the formation of porous zones containing low-density C-S-H phases and localized self-desiccation due to particle aggregation. These mechanisms collectively reduce cement hydration efficiency, ultimately generating micro voids that compromise structural integrity^1^. The SEM images confirmed that EMS at higher dosages (15%) can negatively affect the pore structure by agglomerating fine silica particles, thereby reducing pozzolanic activity. This result aligns with the findings of Beskopylny et al^86^, who noted that excessive micro-silica can lead to poor dispersion and hinder the development of hydrated phases in concrete. The results demonstrated that optimized low-to-medium replacement levels, particularly 5% EMS with 15–25% RHA, significantly enhanced compressive strength compared to the control mix. This improvement was attributed to the high reactivity of EMS at early curing stages and the delayed but sustained pozzolanic contribution of RHA, which becomes more pronounced with extended curing. In contrast, higher substitution levels reduced strength due to binder dilution, excessive silica, particle agglomeration, and increased porosity, thereby disrupting hydration kinetics and weakening the microstructure^87^.

### Comparative water permeability of control and Ternary-based mixes

**Fig. 6:**
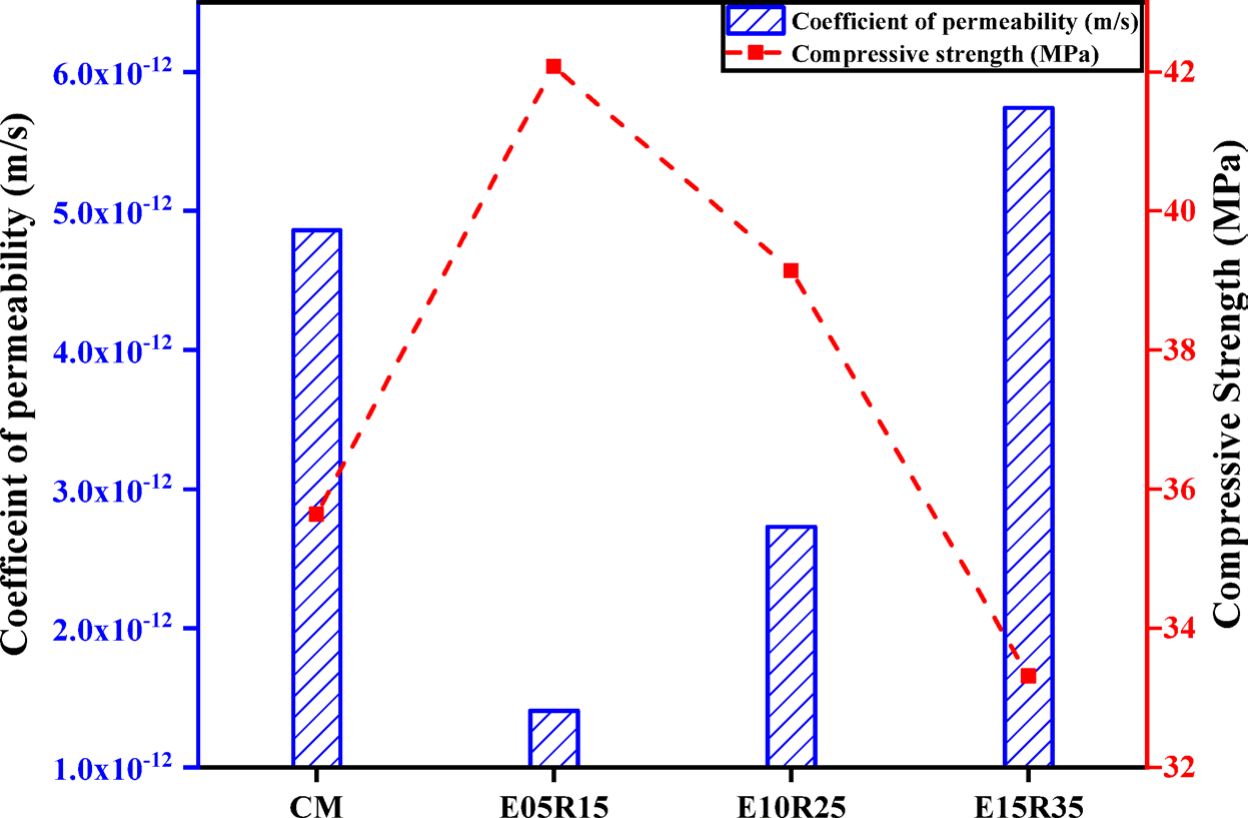
Permeability coefficient of control and ternary mixes at 56 days.

Water permeability is a crucial property affecting the concrete’s long-term durability. Increased water permeability enhances water penetration, thereby increasing the risk of deterioration, particularly when the concrete is exposed to harsh environments such as seawater or industrial water. The resulting higher water intrusion, on the other hand, affects the long-term durability of concrete buildings. Table 8; Fig. 6 show the results of water penetration and the resulting coefficient of permeability. The test results showed that low cement substitution levels (E05R15 and E10R25) showed less water penetration than cement and a ternary mix with a 50% replacement level (E15R35). This reduction in penetration at low replacement dosages may be attributed to filler effects, which fill the gaps between aggregates by incorporating extremely fine particles of EMS and RHA, thereby reducing porosity and preventing water from penetrating the concrete. One of the most prominent implications of the inclusion of pozzolans (such as RHA and SF) is the refinement of the concrete pore structure, which directly leads to a reduction in permeability^88^. These findings were further validated by SEM analysis, which showed that the E05R15 and E10R25 mixes, with well-dispersed particles and refined hydration products, demonstrated improved permeability performance, highlighting their potential for enhanced durability. In contrast, the E15R35 mix, which exhibited unreacted cement and agglomerated micro-silica particles, had higher permeability, confirming that poor dispersion and excessive binder content can reduce water resistance and compromise structural integrity.

**Table 8:** Water permeability and coefficient comparison: control mix vs. ternary mixes.

Mix Designation	Water penetration depth (mm)	Permeability coefficient (m/s)
CM	53.93	4.80 × 10⁻^12^
E05R15	38.10	1.40 × 10⁻^12^
E10R25	47.58	2.72 × 10⁻^12^
E15R35	58.42	5.73 × 10⁻^12^

The experimental and microstructural findings indicate that the interaction between EMS ultra fineness and RHA fineness plays a critical role in the performance of EMS–RHA ternary concrete. Finer particles enhance the packing density and hydration, leading to improved strength and durability. However, at higher dosages, poor dispersion and agglomeration of micro-silica particles reduce the available surface area for pozzolanic reactions, thereby compromising the overall performance of the concrete.

### Statistical analysis and model selection using RSM

The predictive accuracy of the RSM model was evaluated through ANOVA and error analysis, with fit statistics (R^2^, MAE, and RMSE) across all curing intervals (14, 28, and 56 days). These results establish the quadratic model as the most appropriate representation of compressive strength development in EMS–RHA ternary concrete, effectively capturing the nonlinear interactions between binder composition and curing age. Table 9 presents the ANOVA results, demonstrating the significance of the factors and interactions in predicting compressive strength. Model adequacy was verified through coefficient of determination (R²) analysis and p-value assessment across all curing periods (14, 28, and 56 days). The predictive model exhibits statistically significant input-output correlations when satisfying both validation criteria: a coefficient of determination (R²) exceeding 0.80 and a p-value below 0.05^89^. By contrast, linear and 2FI models showed inferior predictive performance, while cubic models did not offer additional accuracy and risked overfitting. Statistical analysis identified quadratic models as optimal for all responses, with significance determined by p-values < 0.05 (95% confidence level) and supported by R² values^90^. These results establish the quadratic model as the most appropriate representation of compressive strength development in EMS–RHA ternary concrete, effectively capturing the nonlinear interactions between binder composition and curing age.

Recent studies corroborate these findings. Afshoon et al^91^. applied RSM to environmentally friendly concretes and reported an R² value of 0.97. In contrast, ANN slightly outperformed RSM (R² = 0.92), highlighting the challenge of fully capturing nonlinear responses. Similarly, Hurtado-Alonso et al^92^. optimized recycled composite concrete using RSM and noted that predictive accuracy decreased at higher replacement levels, underscoring the difficulty of modeling systems with strong nonlinear effects. Compared to these works, the present study’s performance (R² = 0.90, RMSE = 2.78 MPa) is competitive given the greater complexity of EMS–RHA ternary blends, where both replacement level and curing age introduce variability. This demonstrates that RSM remains a robust tool for optimizing eco-efficient concrete mixtures, even in systems with multiple nonlinearities.

**Table 9:** Statistical summary of RSM models for compressive strength at different curing ages.

Source	Compressive strength (14d)			Compressive strength (28d)			Compressive strength (56d)		
	*p*-value	Adjusted *R*^2^	Predicted *R*^2^	*p*-value	Adjusted *R*^2^	Predicted *R*^2^	*p*-value	Adjusted *R*^2^	Predicted *R*^2^
Linear	< 0.0001	0.80	0.77	< 0.0001	0.83	0.82	< 0.0001	0.89	0.88
2FI	0.7153	0.79	0.76	< 0.0001	0.92	0.91	0.0011	0.92	0.91
**Quadratic**	**< 0.0001**	**0.91**	**0.88**	**0.0093**	**0.95**	**0.94**	**0.0451**	**0.93**	**0.91**
Cubic	0.4841	0.90	0.86	0.929	0.95	0.92	0.2342	0.93	0.91
Overall statistical evaluation									
R^2^ 0.90									
MAE 1.73									
RMSE 2.78.									

#### Fit statistics and effect of EMS and RHA on 14-day compressive strength

ANOVA results presented in Table 10 confirm the adequacy of the quadratic model for predicting 14-day compressive strength, with the model achieving statistical significance (F = 97.85, *p* < 0.0001). In the ANOVA analysis, the terms A and B represent EMS and RHA dosages, respectively. The high correlation values (R² = 0.92, adjusted R² = 0.91, predicted R² = 0.88) all exceeded the 0.80 threshold, confirming both the model’s significance and the strong agreement between experimental and predicted outcomes. These metrics collectively demonstrate excellent predictive capability and model fit^93^. The strong agreement between predicted and experimental 14-day compressive strength values (differences < 0.2 MPa) confirms model accuracy. Further validation is provided by the close alignment of R² metrics (difference between predicted and adjusted R² < 0.2), demonstrating both statistical significance and an excellent model fit^94^.

Table 10 shows that EMS dosage (A) exerted the strongest influence (*p* < 0.0001), while the quadratic term B² was also significant, indicating nonlinear effects on early-age strength. Conversely, RHA dosage (B), EMS and RHA interaction (AB), and A², were not significant at 14 days, suggesting that the role of RHA becomes more prominent at later curing ages. The non-significant lack-of-fit statistic (*p* = 0.9927) further validates the model’s adequacy. Collectively, these findings demonstrate that early-age strength development is primarily governed by EMS dosage, with nonlinear contributions further refining the predictive accuracy. Accordingly, the regression equation (Eq. 8) provides a reliable predictive tool for early-age compressive strength in EMS–RHA ternary systems.

8 $${{\mathrm{Y}}_{\mathrm{1}}}{\text{ = + 23}}{\text{.25 - 2}}{\text{.77 A - 0}}{\text{.67 B + 3}}{\text{.29 AB + 0}}{\text{.9884 }}{{\mathrm{A}}^{\mathrm{2}}}_{\text{}}{\text{ - 4}}{\text{.77 }}{{\mathrm{B}}^{\mathrm{2}}}$$

**Table 10:** ANOVA results for compressive strength at 14 days.

ANOVA for the Quadratic Model						
Source	Sum of Squares	df	Mean square	F-value	*p*-value	Remarks
**Model**	239.31	5	53.24	97.85	< 0.0001	significant
A- EMS Dosages	56.27	1	78.49	79.76	< 0.0001	significant
B- RHA Dosages	1.54	1	1.54	5.97	0.1506	not significant
AB	1.73	1	1.73	2.84	0.1294	not significant
A^2^	1.36	1	1.36	1.61	0.1767	not significant
B^2^	4.98	1	4.98	9.30	0.0131	significant
Lack of fit	0.51	6	0.08	0.12	0.9927	not significant
**Fit Statistics**						
R^2^ 0.92 Remarks: The quadratic Model is significant to proceed with the design.						
Adjusted R^2^ 0.91						
Predicted R^2^ 0.88						
Adeq. Precision 23.79.						

**Fig. 7:**
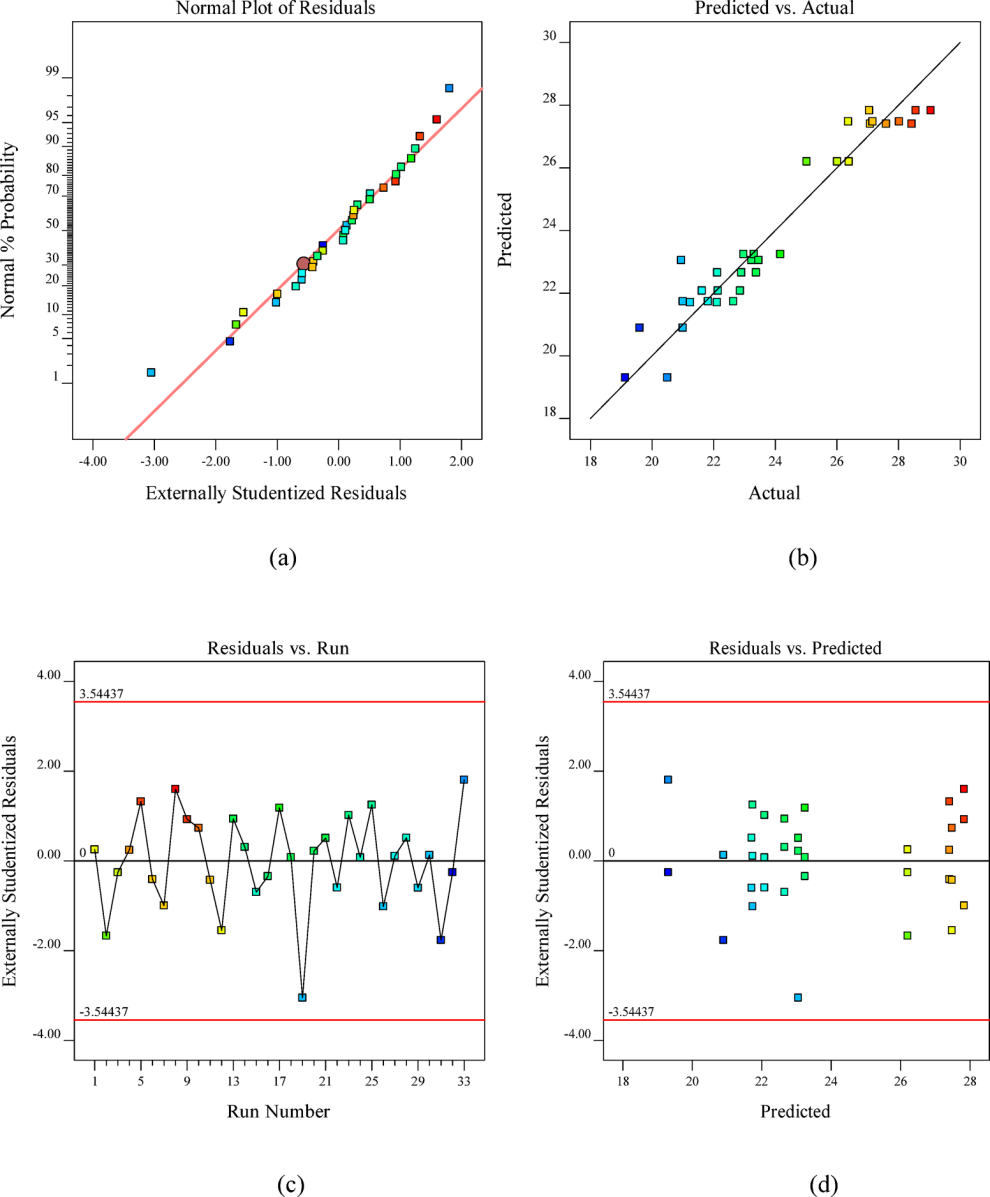
Validation plots for the 14-day RSM Model: **(a)** normal probability of residuals, **(b)** predicted vs. actual values, **(c)** residuals vs. predicted values, and **(d)** residuals vs. run number.

In addition to ANOVA, graphical diagnostics supplemented quantitative analyses, with probability plots verifying residual normality and scatter plots illustrating the experimental-predicted correlation, as shown in Fig. 7. The plots provide critical visual confirmation of model adequacy, confirming the suitability of quadratic models for the experimental data^95^. The normal probability plot of residuals (Fig. 7(a)) shows that the data points closely follow a straight line, indicating that the residuals are normally distributed. Figure 7(b) illustrates the predicted versus actual values, with data points closely following the 45° reference line, thereby confirming strong correlation and high predictive accuracy of the model. The residuals-versus-predicted plot (Fig. 7(c)) shows random scatter within the defined confidence limits, indicating the absence of heteroscedasticity and validating the reliability of the predictions. This random distribution demonstrates the absence of heteroscedasticity and further supports the model’s predictive reliability^89^. Finally, the residuals versus run order plot **(**Fig. 7(d)) displays a random distribution without systematic trends, confirming the independence of residuals and ruling out time-related bias in the experimental runs. Collectively, these diagnostic plots provide strong visual evidence supporting the adequacy and robustness of the quadratic regression model^96^.

**Fig. 8:**
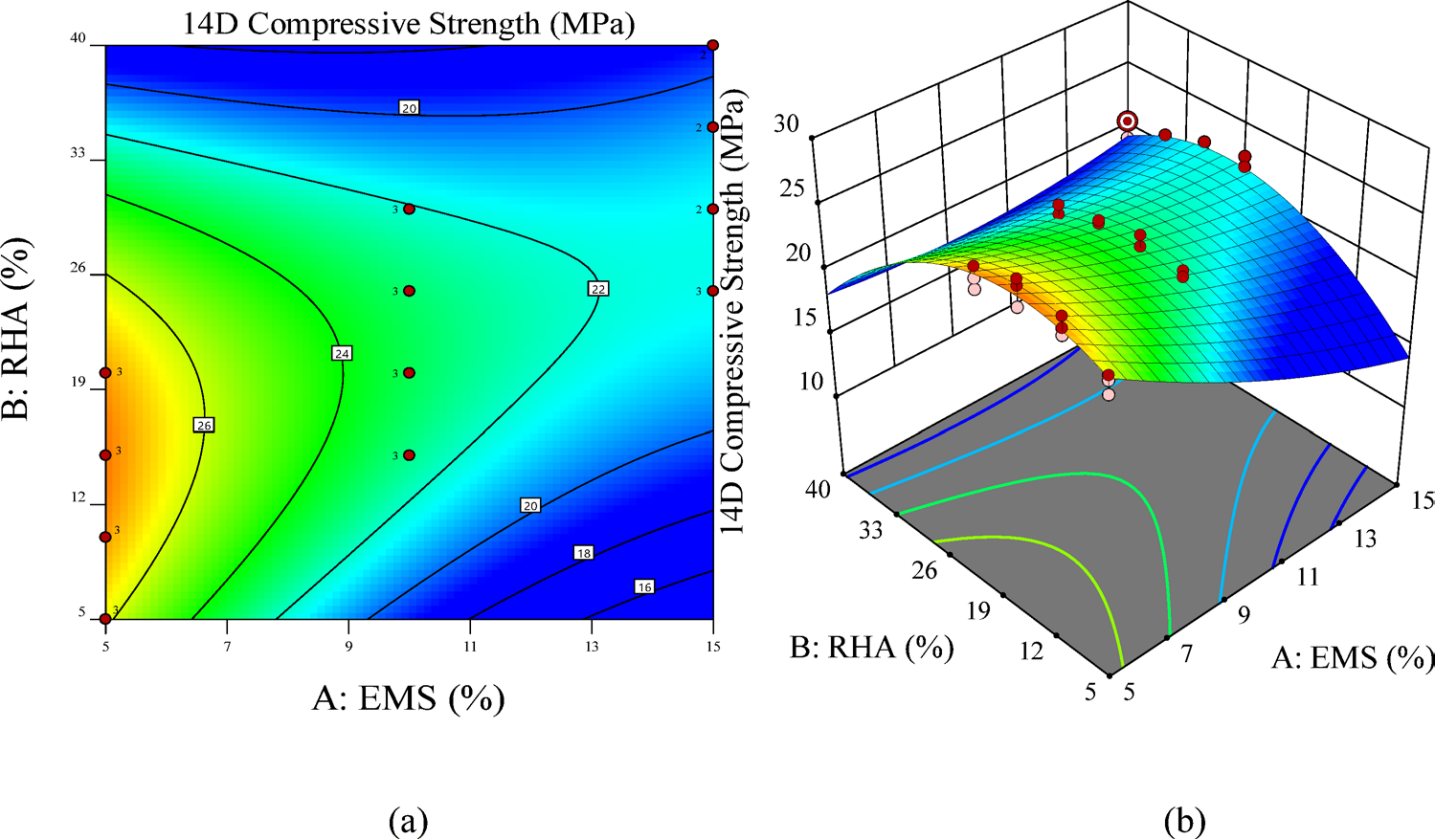
RSM visualization for 14-day compressive strength: **(a)** 2D contour plot showing EMS–RHA interaction effects and **(b)** 3D response surface illustrating the combined influence of both variables.

Figure 8 illustrates the combined effect of EMS and RHA dosages on 14-day compressive strength through a 2D contour (Fig. 8 (a)) and 3D surface plots (Fig. 8 (b)). The contour map highlights distinct dosage-dependent trends, where EMS contributes most effectively to early-age strength at lower incorporation levels (5–10%). Beyond this range, the marginal benefit diminishes due to particle agglomeration and dilution effects, as seen validated by SEM scans. In contrast, RHA demonstrates improved performance at moderate replacement levels (15–30%), attributed to its pozzolanic reactivity becoming more effective after initial hydration.

The 3D response surface further confirms these patterns, showing a peak strength response within the interaction region of low EMS (5–10%) and medium RHA (15–25%), consistent with the statistical significance of EMS dosage (*p* < 0.0001) and quadratic effects identified in ANOVA (Table 10). At higher EMS–RHA combinations, compressive strength decreases, reflecting the nonlinear relationship captured by the quadratic model. These visualizations reinforce the conclusion that early-age strength is optimized by low EMS and medium RHA dosages, aligning with both experimental outcomes and model predictions.

#### Fit statistics and effect of EMS and RHA on 28-day compressive strength

ANOVA results are presented in Table 11, demonstrating the statistical significance of the 28-day compressive strength Model. Significant terms (*p* < 0.05) and a high F-value (105.42) confirm the Model’s adequacy. EMS dosage (A), along with the quadratic terms A² and B², were identified as significant contributors (*p* < 0.05), whereas RHA dosage (B) and the interaction term (A×B) were not statistically significant. These results indicate that EMS exerts a strong linear influence on compressive strength. Conversely, the linear effect of RHA (B) and the interaction term (AB) were not significant, suggesting that RHA primarily influences 28-day strength through its quadratic response rather than direct or interactive effects. The Model exhibited excellent fit statistics (R² = 0.96, adjusted R² = 0.95, predicted R² = 0.94), and the lack-of-fit test was non-significant (F = 0.12, *p* = 0.9934), confirming that the quadratic form adequately represents the experimental data^81^. The derived quadratic regression equation (Eq. 9) enables the prediction of compressive strength (Y_2_) across variations in these parameters.

9 $${{\mathrm{Y}}_{\mathrm{2}}}\,{\mathrm{=}}\,{\mathrm{+}}\,{\mathrm{32}}{\mathrm{.43-3}}{\text{.59 A-0}}{\text{.0881 B}}\,{\mathrm{+}}\,{\mathrm{3}}{\mathrm{.09AB-}}\,{\mathrm{2}}{\text{.35 }}{{\mathrm{A}}^{\mathrm{2}}}{\mathrm{-}}\,{\mathrm{4}}{\text{.02 }}{{\mathrm{B}}^{\mathrm{2}}}$$

These results show that EMS dosage exerts the strongest linear effect at 28 days, while RHA primarily influences performance through nonlinear effects, reinforcing the importance of optimizing combined replacement levels to maximize performance.

**Table 11:** ANOVA results for compressive strength at 28 days.

ANOVA for the Quadratic Model						
Source	Sum of Squares	df	Mean square	F-value	*p*-value	Remarks
**Model**	274.63	5	54.93	105.45	< 0.0001	significant
A- EMS Dosages	69.82	1	69.82	134.04	< 0.0001	significant
B- RHA Dosages	0.0227	1	0.0227	0.043	0.8367	not significant
AB	1.21	1	1.21	2.32	0.1427	not significant
A^2^	6.00	1	6.00	11.52	0.0027	significant
B^2^	2.83	1	2.83	5.44	0.0297	significant
Lack of fit	0.51	6	0.08	0.12	0.9934	not significant
**Fit Statistics**						
R^2^ 0.96 Remarks: The quadratic Model is significant to proceed with the design.						
Adjusted R^2^ 0.95						
Predicted R^2^ 0.94						
Adeq. Precision 25.91.						

**Fig. 9:**
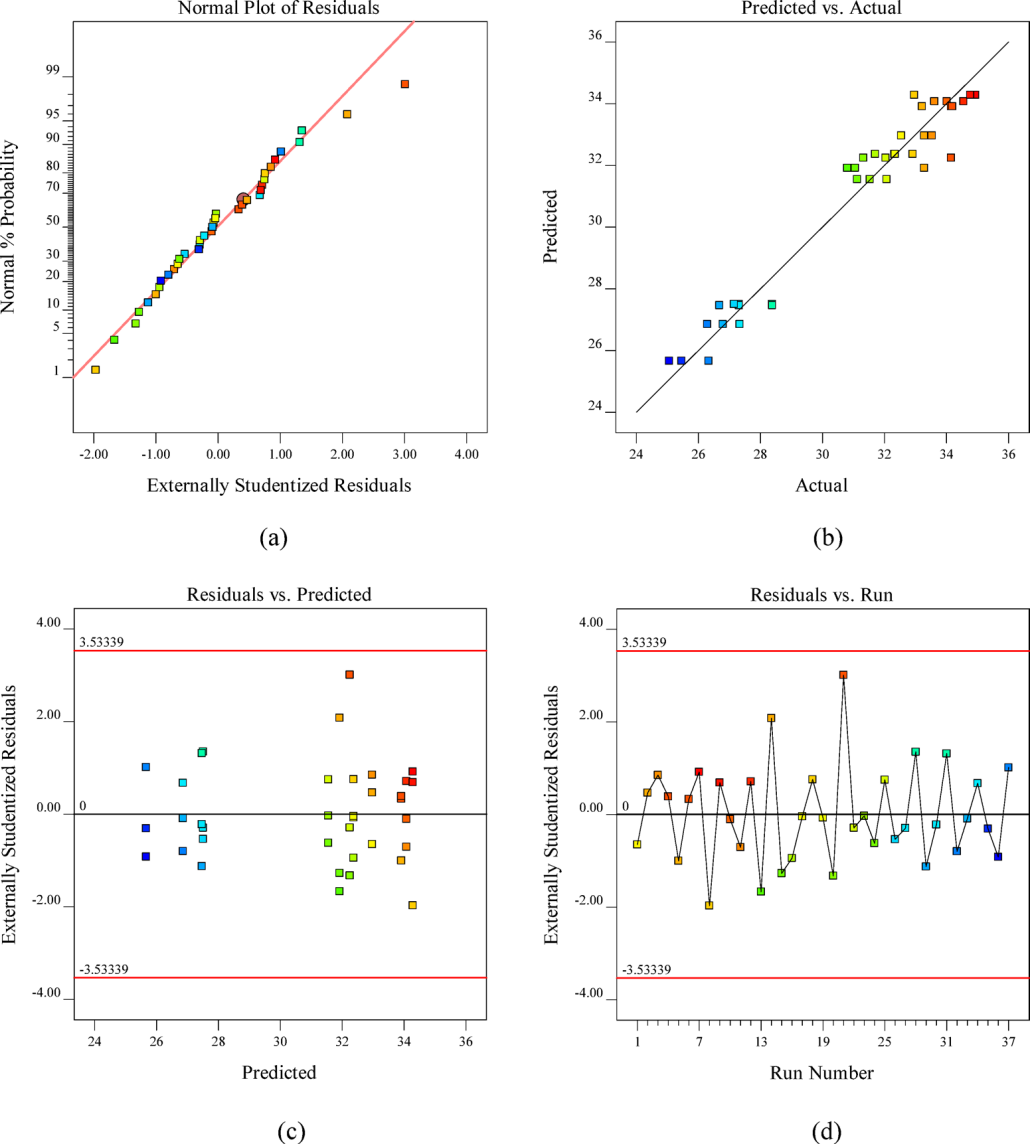
Validation plots for the 28-day RSM model: **(a)** normal probability of residuals, **(b)** predicted vs. actual values, **(c)** residuals vs. predicted values, and **(d)** residuals vs. run number.

In addition to ANOVA, graphical diagnostics supplemented quantitative analyses, with probability plots verifying residual normality and scatter plots illustrating the experimental-predicted correlation, as shown in Fig. 9. At 28 days, the adequacy of the quadratic regression Model was further validated through residual diagnostics. Figure 9(a) shows that the normal probability plot confirms the normal distribution of residuals, while Fig. 9(b) demonstrates close alignment along the 45° line, indicating excellent predictive accuracy. Figure 9(c) shows the residuals-versus-predicted plot, which displays random scatter within the confidence limits, confirming constant variance and the absence of heteroscedasticity. Similarly, Fig. 9(d) shows the residuals versus run plot, which reveals random fluctuations, indicating independence of the residuals and no evidence of time-related bias^96^. Collectively, these diagnostic plots reinforce the ANOVA findings in Table 11, which establish that the quadratic regression model provides a statistically robust and reliable prediction of 28-day compressive strength.

**Fig. 10:**
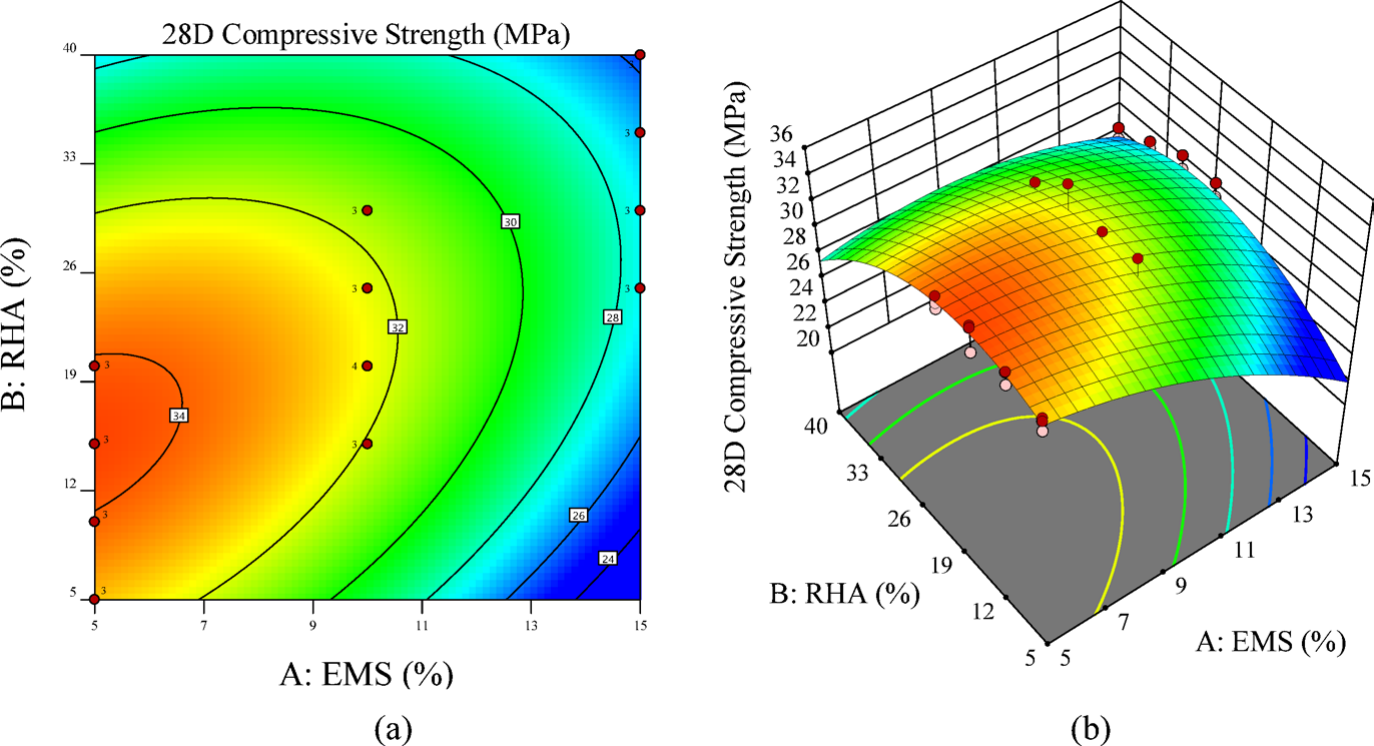
RSM visualization for 28-day compressive strength: **(a)** contour plot and **(b)** 3D surface plot showing EMS–RHA interaction effects on strength development.

Figure 10 presents the 2D contour and 3D surface plots illustrating the combined effects of EMS and RHA on the 28-day compressive strength of ternary concrete mixtures. The response surfaces reveal clear dosage-dependent behaviors, with EMS and RHA exerting distinct yet complementary influences. EMS provided its maximum strength enhancement at relatively low incorporation levels (5–10%), demonstrating its high reactivity and efficiency in refining the pore structure during the early hydration stage. In contrast, RHA exhibited peak contributions at intermediate dosages (15–30%), which aligns with its pozzolanic nature, as sufficient curing time is required for RHA-silica to effectively react with portlandite and form additional C-S-H gel.

The contour plots highlight that optimal strength is achieved at low EMS with moderate RHA levels. This balance highlights the synergy between highly reactive EMS and the delayed pozzolanic reactivity of RHA, ensuring both strength enhancement and sustainability benefits. At higher replacement levels, however, the surfaces indicate a decline in compressive strength, attributed to binder dilution and excessive silica, which leads to agglomeration and incomplete hydration.

These findings corroborate the ANOVA results in Table 11, which identified EMS dosage as the dominant linear factor and both EMS and RHA quadratic terms (A², B²) as significant contributors. Collectively, the results emphasize that balancing EMS and RHA is crucial for maximizing compressive strength while ensuring sustainability benefits.

#### Fit statistics and effect of EMS and RHA on 56-day compressive strength

Table 12 presents the most statistically significant ANOVA terms used in developing the quadratic regression model for predicting 56-day compressive strength. The quadratic ANOVA model for 56-day compressive strength was highly significant (F = 105.49, *p* < 0.0001), supported by excellent fit statistics (R² = 0.93, Adjusted R² = 0.93, Predicted R² = 0.91). EMS dosage (A) remained the most influential factor (*p* < 0.0001), while RHA dosage (B) also became significant (*p* = 0.0107), reflecting its pozzolanic activity at later curing stages. Quadratic terms A² and B² were both significant (*p* < 0.05), confirming nonlinear behavior. The interaction term AB was not significant, suggesting additive rather than synergistic contributions of EMS and RHA. The lack-of-fit test was non-significant (*p* = 0.58), indicating that the model adequately captured the variability in the experimental data. These results highlight that EMS contributes significantly to strength across all curing ages, whereas RHA becomes more impactful at extended curing durations due to its slower reactivity. The derived quadratic regression equation (Eq. 10) enables the prediction of compressive strength (Y_3_) across variations in these parameters.

10 $${{\mathrm{Y}}_{\mathrm{3}}}\,{\mathrm{=}}\,{\mathrm{+}}\,{\mathrm{38}}{\mathrm{.82-3}}{\text{.30 A-1}}{\text{.27 B}}\,{\mathrm{+}}\,{\mathrm{4}}{\text{.23 AB-}}\,{\mathrm{1}}{\text{.89 }}{{\mathrm{A}}^{\mathrm{2}}}{\mathrm{-}}\,{\mathrm{4}}{\text{.59 }}{{\mathrm{B}}^{\mathrm{2}}}$$

This predictive equation provides a reliable tool for optimizing ternary binder proportions to achieve durable, high-strength, and sustainable concrete at advanced curing ages.

**Table 12:** ANOVA and fit statistics of compressive strength at 56 days.

ANOVA for the Quadratic Model						
Source	Sum of Squares	df	Mean square	F-value	*p*-value	Remarks
**Model**	471.12	5	94.22	105.49	< 0.0001	significant
A- EMS Dosages	86.87	1	86.87	97.25	< 0.0001	significant
B- RHA Dosages	6.52	1	6.52	7.30	0.0107	significant
AB	3.21	1	3.21	3.60	0.0664	Not significant
A^2^	5.35	1	5.35	5.99	0.0197	significant
B^2^	5.56	1	5.56	6.22	0.0177	Significant
Lack of fit	4.55	6	0.75	0.82	0.58	Not significant
**Fit Statistics**						
R^2^ 0.93 Remarks: The quadratic Model is significant to proceed with the design.						
Adjusted R^2^ 0.93						
Predicted R^2^ 0.91						
Adeq. Precision 26.82.						

**Fig. 11:**
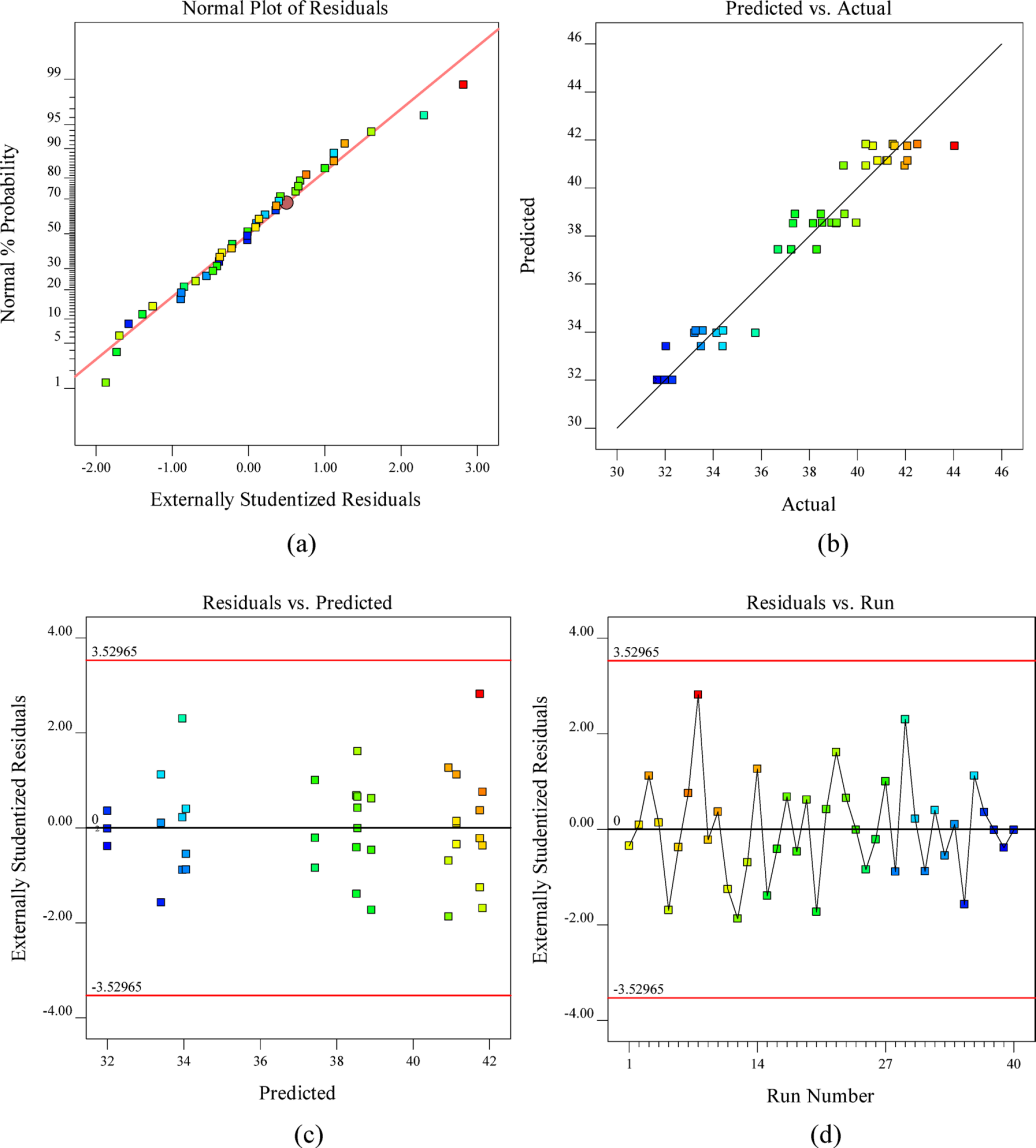
Validation plots for the 56-day RSM Model: **(a)** normal probability of residuals, **(b)** predicted vs. actual values, **(c)** residuals vs. predicted values, and **(d)** residuals vs. run number.

At 56 days, the adequacy of the quadratic regression model was confirmed through residual diagnostics Fig. 11. The plots provide critical visual confirmation of model adequacy, confirming the suitability of quadratic models for the experimental data^95^. Figure 11(a) presents the normal probability plot for the 56-day compressive strength model, where the linear alignment of the data points confirms the normal distribution of the residuals. The close agreement between experimental and predicted values is evident from the minimal deviation of the points from the reference line. Figure 11(b) shows the close clustering of points along the 45° reference line, indicating strong agreement between the experimental and predicted values and confirming the model’s accuracy. Figure 11(c) The residuals versus predicted plot indicated random scatter within the confidence limits, validating constant variance of residuals. This pattern confirms the model’s predictive accuracy and the homoscedasticity of the residuals^89^. Figure 11(d) presents the residuals versus run order plot, where the random sinusoidal distribution of data points within the confidence boundaries (red lines) confirms the absence of systematic drift or time-dependent bias in the model^96^. Collectively, these diagnostics, combined with the non-significant lack-of-fit test, establish the robustness of the quadratic model for predicting long-term compressive strength.

**Fig. 12:**
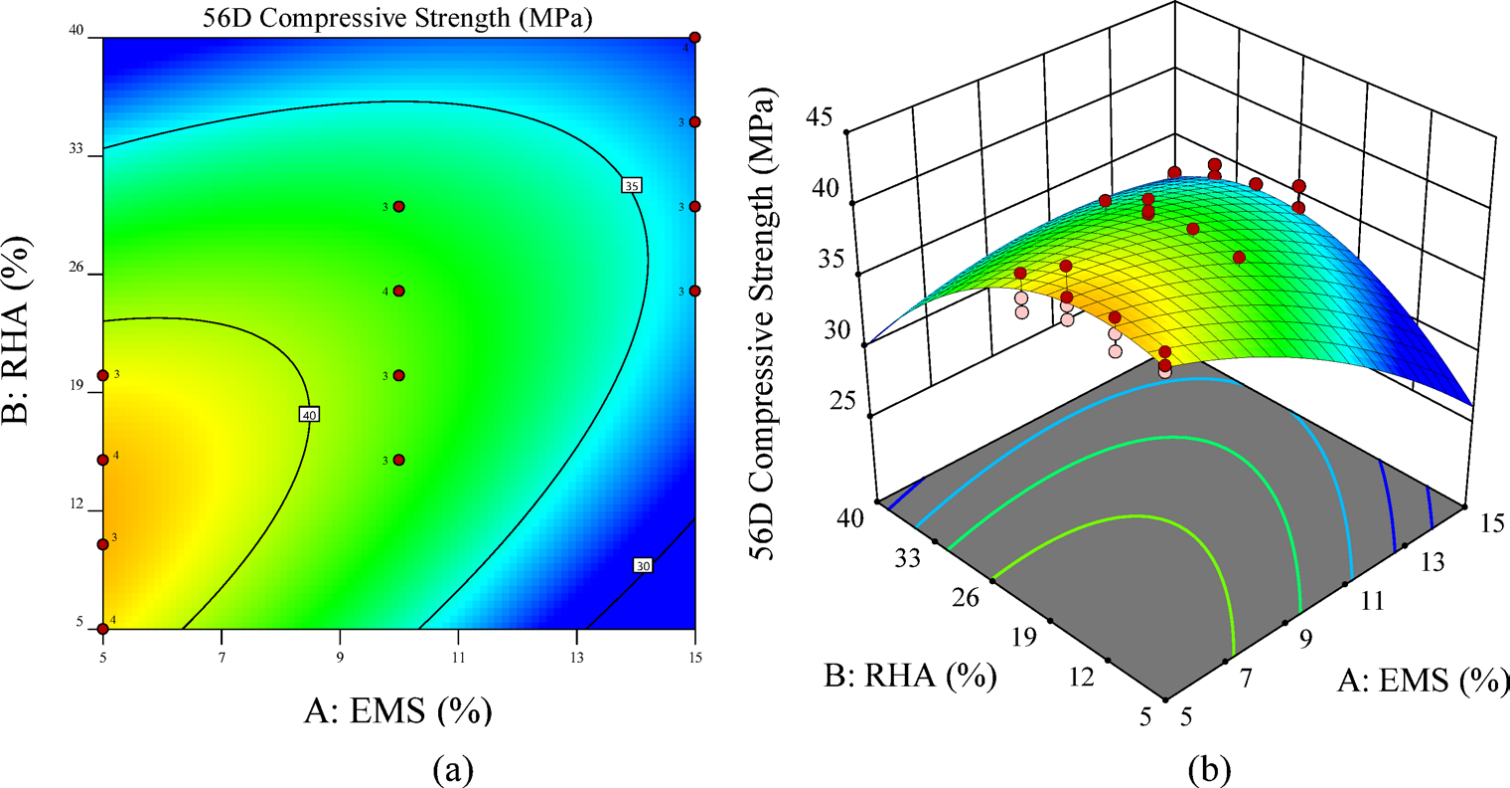
RSM visualization for 56-day compressive strength: **(a)** 2D contour plot and **(b)** 3D surface plot illustrating EMS–RHA interaction patterns at later ages.

Figure 12 presents the 2D contour and 3D surface plots illustrating the combined effects of EMS and RHA on the 28-day compressive strength of ternary concrete mixtures. The response surfaces reveal clear dosage-dependent behaviors, with EMS and RHA exerting distinct yet complementary influences. Maximum strength enhancement was achieved at relatively low incorporation levels (5–10%), confirming its high reactivity and efficiency in refining pore structure during early hydration. In contrast, RHA exhibited peak contributions at intermediate dosages (15–30%), which aligns with its pozzolanic nature, as sufficient curing time is required for RHA-silica to effectively react with portlandite and form additional C-S-H gel.

The contour plots show that optimal strength performance is achieved when low EMS content is combined with moderate RHA replacement, confirming the synergy among ultrafine reactive silica (EMS), the delayed pozzolanic nature, and sustainable RHA. At higher replacement levels, however, the surfaces indicate a decline in compressive strength, attributed to binder dilution and excessive silica, which leads to agglomeration and incomplete hydration.

These observations are consistent with the ANOVA results (Table 12), which identified EMS dosage as the dominant linear factor and both EMS and RHA quadratic terms (A², B²) as significant contributors to long-term strength. Collectively, the results emphasize that a balanced incorporation of EMS and RHA is essential to maximize compressive strength while ensuring sustainability benefits.

Statistical significance analysis using ANOVA confirmed that the quadratic Model is highly significant for predicting compressive strength at all curing ages, with Model p-values < 0.001 and F-values far exceeding unity. EMS dosage (factor A) exhibited a statistically significant effect on early-age strength (14 and 28 days), as indicated by *p* < 0.0001, while RHA dosage (factor B) showed a milder but still meaningful contribution at later ages. Significant interaction terms (A² and AB) further verified the presence of non-linear relationships between the variables.

#### Optimization of factors using RSM

The compressive strength of cement composites at different curing ages is strongly influenced by the binder composition, particularly the proportions of EMS and RHA. In this study, EMS and RHA dosages were selected as independent variables, while the 14-, 28-, and 56-day compressive strengths were treated as response variables (Table 13). A multi-objective optimization approach was implemented using an RSM framework to identify the optimal factor combinations that maximize strength development across curing periods. The optimization criteria were defined as follows: EMS dosage constrained to 5–15%, RHA dosage to 5–40%, and compressive strength at 14, 28, and 56 days to be maximized. This framework ensured that the solutions balanced both early- and long-term performance while remaining within practical and sustainable replacement ranges. The results of the optimization analysis provide practical guidelines for achieving high-performance ternary cement composites by leveraging the synergistic contributions of EMS (rapid early-age reactivity) and RHA (delayed pozzolanic activity).

**Table 13:** RSM optimization criteria for EMS, RHA, and compressive strength outputs.

Responses	Criteria
EMS (%)	In the range (5–15%)
RHA (%)	In the range (5–40%)
14-day CS	Maximize
28-day CS	Maximize
56-day CS	Maximize

**Fig. 13:**
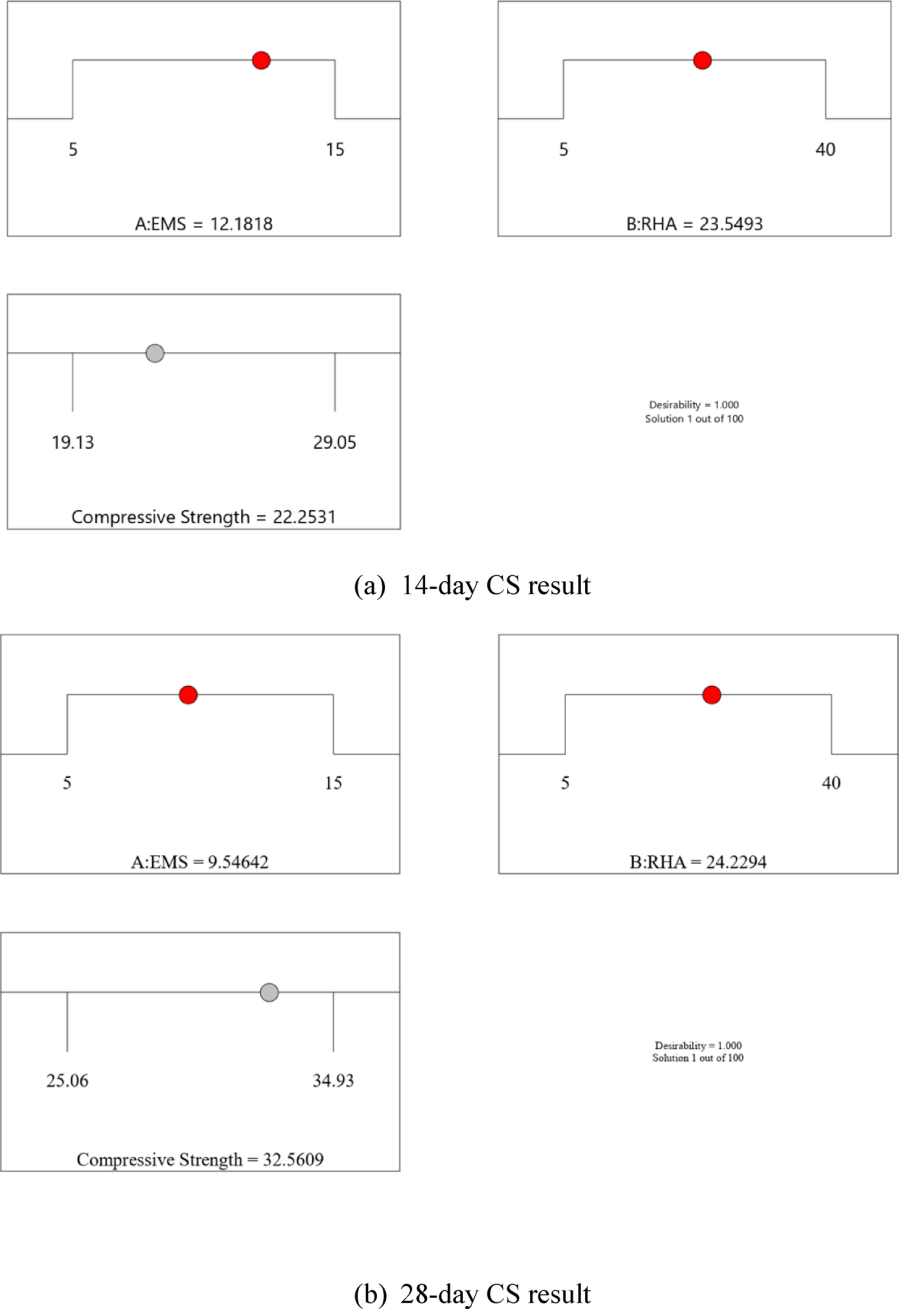

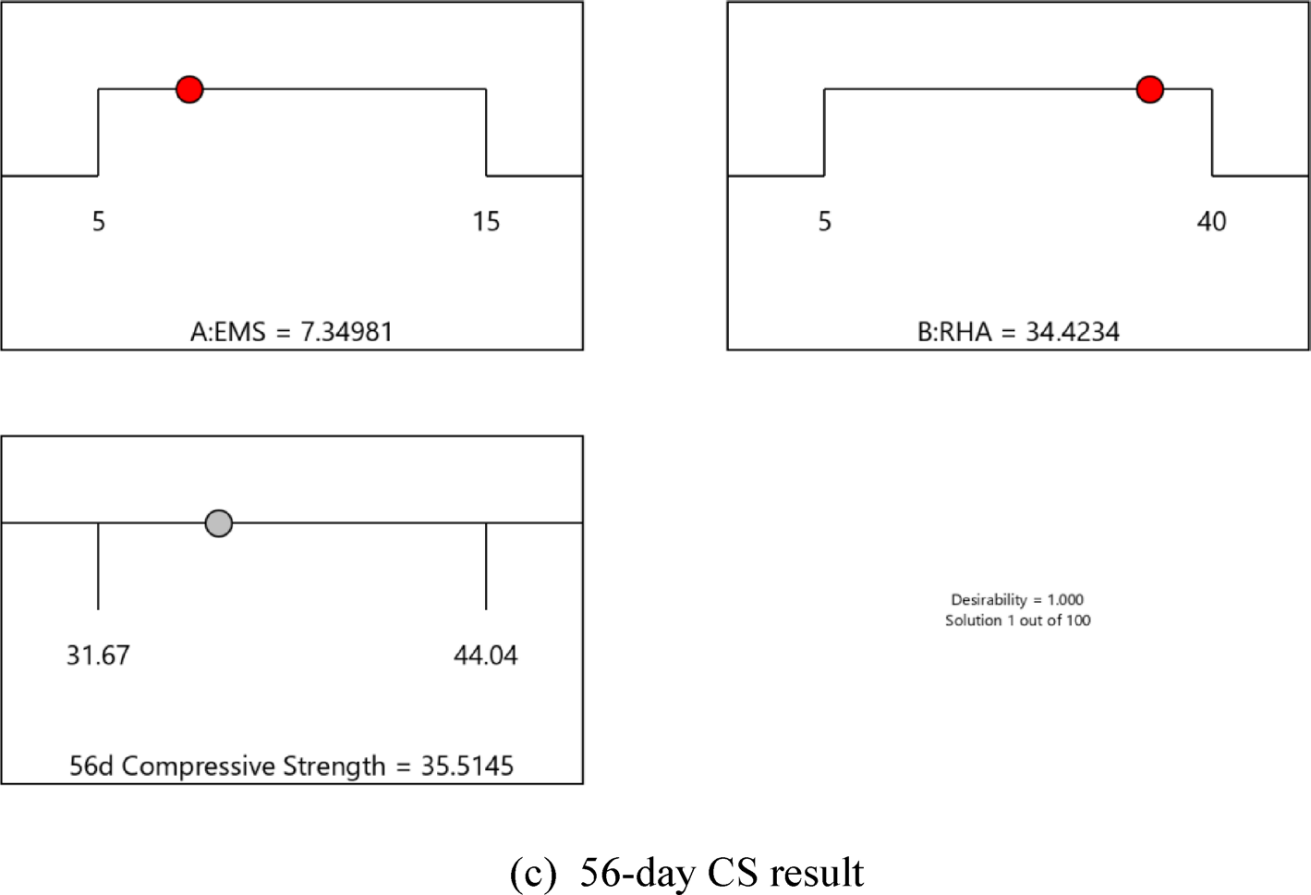
RSM numerical optimization outputs showing optimum EMS and RHA contents along with predicted compressive strength development at 14, 28, and 56 days.

An optimization ramp was developed to evaluate the influencing factors (EMS and RHA) and the corresponding response variables (compressive strength at 14, 28, and 56 days), as illustrated in Fig. 13. The red markers represent the optimal input factor levels, while the grey markers indicate the optimized compressive strength outcomes. The Model identified optimal dosages of EMS (12.18%) and RHA (23.55%), yielding a predicted 14-day CS compressive strength of 22.25 MPa. The optimal factor combination was EMS (9.55%) and RHA (24.23%), resulting in a predicted compressive strength of 32.56 MPa for 28-day CS. Optimum values were achieved at EMS (7.35%) and RHA (34.42%), with a predicted compressive strength of 35.51 MPa (Table 14). All solutions achieved a desirability index of 1.000, confirming that the optimization framework successfully balanced the factor ranges to maximize compressive strength at each curing stage. The results clearly show a dosage-dependent trend, with EMS contributing strongly at early ages and RHA exerting greater influence at later curing periods. This demonstrates the complementary roles of EMS and RHA in enhancing both short-term and long-term mechanical performance of ternary cement composites.

**Table 14:** Optimal EMS–RHA dosages and predicted compressive strengths at different curing ages.

Curing Age (days)	Optimal EMS (%)	Optimal RHA (%)	Predicted CS (MPa)	Desirability
14	12.18	23.55	22.25	1.000
28	9.55	24.29	32.56	1.000
56	7.35	34.42	35.51	1.000

To confirm the optimized results, additional experiments were conducted using the ideal mixing parameters for the concrete mixtures. The agreement between predicted and experimental values was assessed by calculating the relative error using Eq. (11), thereby verifying the established relationship.

11 $$\:\:Relative\:Error\:\left(\mathrm{\%}\right)\:=\:\frac{Experimental\:result-predicted\:result}{Experimental\:result}*100$$

The experimental validation of the optimal parameters derived from RSM is presented in Table 15. A relative error below 5% confirms strong agreement between the RSM-predicted values and the actual experimental results, demonstrating the reliability of the optimization approach^97^.

**Table 15:** Validation of predicted versus experimental compressive strength results.

Description	Compressive strength (MPa)		
	14 Days	28 Days	56 Days
Predicted Value	22.25	32.59	35.51
Experimental Value	23.08	31.27	36.68
Relative Error (%)	3.59	4.22	3.18

### Statistical analysis of the ANN model

In this study, a comprehensive database was compiled from experimental data to examine the influence of EMS, RHA, curing age, and other mix parameters on compressive strength (CS). Prior to Model development, a statistical assessment of the selected variables was performed to characterize their distribution and variability. The dataset included key mixture variables, such as cement, extracted micro silica (EMS), rice husk ash (RHA), and superplasticizer (SP) content, as well as curing age (Age). Additionally, the quantities of fine aggregate, coarse aggregates 20 mm and 10 mm, and water were kept constant at 700.0 kg/m³, 682.0 kg/m³, 418.0 kg/m³, and 157.5 kg/m³, respectively. The descriptive statistics, including mean, median, mode, standard deviation, variance, skewness, and kurtosis, are presented in Table 16, providing a clear indication of the spread and central tendency of each parameter. These statistical measures were used to confirm the dataset’s representativeness and ensure that the selected variables capture the experimental variability required for reliable modeling. Therefore, an ANN was adopted as a data-driven predictive Model to estimate the compressive strength of ternary-based concrete incorporating EMS and RHA at varying percentages.

**Table 16:** Descriptive statistics for the experimental dataset, including cement, EMS, RHA, SP, Age, and CS.

	Cement (kg/m³)	EMS (kg/m³)	RHA (kg/m³)	SP (%)	Age (Days)	CS (MPa)
**Mean**	314.7	41.5	93.8	1.3	32.7	30.7
**Median**	315.0	45.0	90.0	1.3	28.0	31.5
**Mode**	337.5	22.5	67.5	1.3	14.0	33.3
**Standard Deviation**	70.9	21.4	51.3	0.1	17.5	6.5
**Sample Variance**	5020.0	459.3	2635.1	0.0	307.5	41.6
**Kurtosis**	−0.8	−1.0	−0.7	0.5	−1.5	−1.0
**Skewness**	0.2	−0.2	−0.1	−0.7	0.4	0.1
**Minimum**	198.5	0.0	0.0	1.0	14.0	18.6
**Maximum**	450.0	67.5	184.0	1.4	56.0	44.0

#### Performance of the developed ANN model

The artificial neural network comprises three layers: an input layer, a hidden layer, and an output layer. The Levenberg-Marquardt backpropagation algorithm was used to develop an artificial neural network. The ANN Model was developed using 117 experimentally obtained data points. This proprietary dataset was necessary because no pre-existing data on ternary-concrete mixtures incorporating these specific materials (EMS and RHA) are available in the published literature. Initially, the ANN algorithm was used to randomly split the database into 80% for training and 20% for testing to avoid bias. The ANN was trained exclusively on the training set, and the held-out test set was used for final performance assessment. The ANN input data were used in their original, unscaled units. Data normalization was not adopted because the study aimed to preserve the true physical and percentage-biased significance of the input parameters (EMS, RHA, and curing age). This approach was adopted to maintain the Model’s physicochemical interpretability, as the primary objective included a sensitivity analysis to determine the real-world influence of each parameter^98,99^. This approach empowers engineers to optimize concrete mixes by clarifying the practical influence of each constituent, providing actionable insight beyond mere numerical predictions^100^. The use of raw data ensures a direct correspondence between the Model’s connection weights and the actual experimental variables, a necessity for generating domain-relevant insights^101,102^. The Model’s high performance confirms that this method did not hinder its predictive capability. The input layer, comprised of the influential variables, was passed to the hidden layer. Each connection between two neurons is accompanied by an associated weight that stores the relative importance of an input to the predicted output. These weighted inputs are summed with a bias term, passed through the activation function, and then passed onto the subsequent layer. The training procedure continually updates the weights and biases until the Model has a lower error, as quantified by convergence metrics or a finite number of training epochs. The mathematical equations involved in these steps are given in Eqs. 12–14.

12 $$\:{s}_{j}={\sum\:}_{i=1}^{n}{x}_{ij}{w}_{ij}+{b}_{jj},\:j=1,\:2,\:3,\:\dots\:,\:n$$

13 $$\:{f}_{c}={\sum\:}_{j=1}^{k}{y}_{kj}{w}_{kj}+{b}_{kk},\:k=1,\:2,\:3,\:\dots\:,\:n$$

14 $$\:{f}_{sig}=\frac{1}{1-{e}^{-{s}_{j}}\:},\:\:\:j=1,\:2,\:3,\dots\:,\:n$$

where, $\:{s}_{j}$ and $\:{f}_{c}$ are the input and output neurons, respectively, while $\:{x}_{ij}$ and $\:{y}_{kj}$ are the input and output variables, respectively, whereas, $\:{b}_{jj}$ and $\:{b}_{kk}$ are the corresponding basis values for each neuron and $\:{w}_{ij}$ and $\:{w}_{kj}$and these are the weights of the input and output variables.

**Fig. 14:**
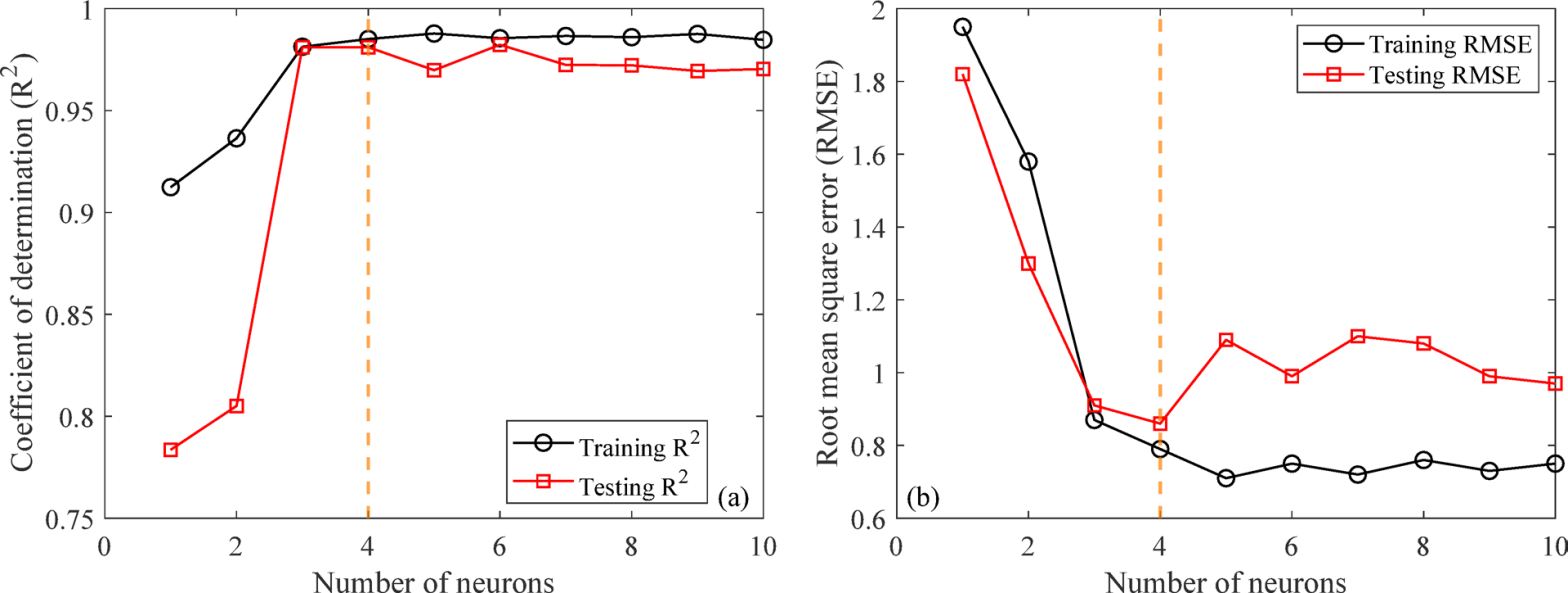
Coefficient of determination (R²) and Root Mean Square Error (RMSE) for training and testing datasets.

Several ANN models were developed by varying the number of hidden-layer neurons from 1 to 10. The comparative performance of these models is shown in Table 17, which depicts the impact of neuron count on prediction accuracy. Models with few neurons had inferior predictive capacity because their low complexity was insufficient to learn the underlying patterns in the data. Conversely, excessively high neuron counts led to overfitting, where the Model performed well during training but showed a dramatic decline in accuracy on the test dataset. It is clear that overfitted models generalize poorly, primarily because they memorize the training data rather than learn patterns that apply to unseen scenarios, as shown in Fig. 14.

**Table 17:** ANN model performance metrics for different neuron configurations.

Layers	Neurons	Training					Testing				
		R	R^2^	RMSE	MAE	RAE	R	R^2^	RMSE	MAE	RAE
1	1	0.9552	0.9124	1.951	1.468	0.260	0.8852	0.7836	1.815	1.380	0.313
	2	0.9677	0.9364	1.583	1.138	0.213	0.8973	0.8051	1.301	1.191	0.228
	3	0.9906	0.9813	0.870	0.699	0.131	0.9905	0.9811	0.912	0.765	0.129
	***4***	***0.9925***	***0.9851***	***0.793***	***0.650***	***0.119***	***0.9905***	***0.9811***	***0.860***	***0.704***	***0.125***
	5	0.9939	0.9878	0.714	0.589	0.106	0.9848	0.9698	1.085	0.896	0.173
	6	0.9927	0.9855	0.752	0.621	0.118	0.9911	0.9823	0.990	0.770	0.126
	7	0.9933	0.9866	0.724	0.591	0.114	0.9861	0.9724	1.099	0.903	0.159
	8	0.9930	0.9860	0.755	0.613	0.113	0.9860	0.9722	1.077	0.942	0.167
	9	0.9938	0.9876	0.732	0.597	0.106	0.9846	0.9694	0.991	0.774	0.162
	10	0.9923	0.9847	0.748	0.609	0.108	0.9851	0.9704	0.972	0.822	0.171

**Fig. 15:**
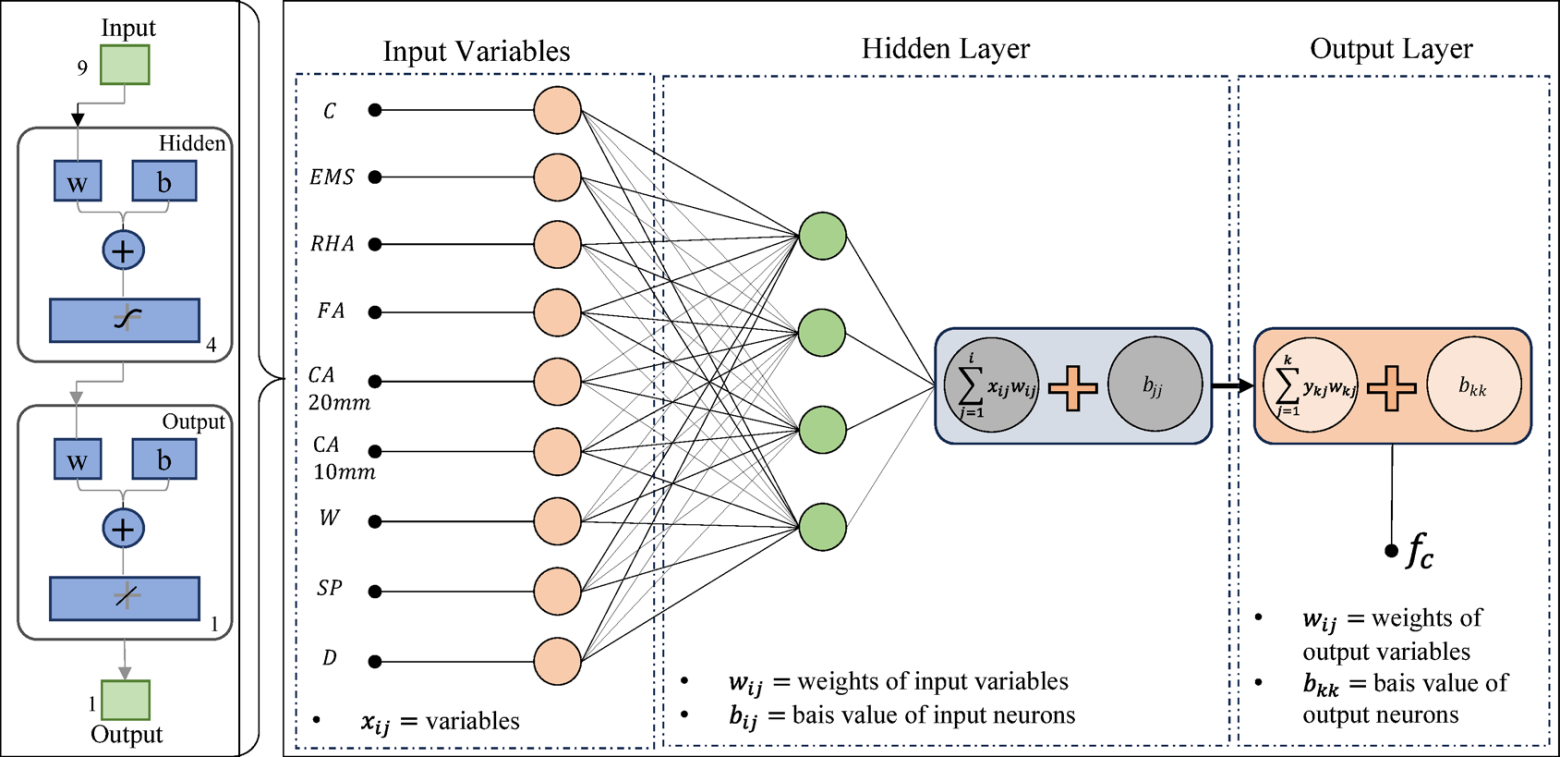
Architecture of the proposed ANN Model for compressive strength prediction.

The predictive Model was constructed using a single-layer perceptron architecture, with hyperparameters optimized via systematic trial-and-error experimentation. The proposed neural network is shown in Fig. 15. The predictive accuracy of the developed computational models was rigorously assessed using multiple statistical indicators: the R², MAE, and RMSE.

The correlation coefficient (R) analysis showed strong predictive performance, with values of 0.9851 and 0.9811 for the training and validation datasets, respectively. These high correlation values demonstrate the Model’s effectiveness in capturing the relationship between experimental and predicted compressive strength values. Error analysis yielded MAEs of 0.650 (Training dataset) and 0.70 (Testing dataset). The lower MAE values quantify the average absolute deviation between Model predictions and experimental measurements, with lower values indicating superior predictive performance. The marginal difference between training and validation MAE values suggests good Model generalization with minimal overfitting. The results from the 4th neuron were selected based on the lowest error. The predictive Model’s performance was further evaluated using root mean square error (RMSE) analysis, which quantifies the standard deviation of prediction errors. The analysis yielded RMSE values of 0.793 MPa for the training dataset and 0.860 MPa for the validation dataset. These results indicate that the Model achieved slightly better predictive accuracy on the training set (lower RMSE). Both datasets showed comparable error ranges, suggesting consistent performance.

**Fig. 16:**
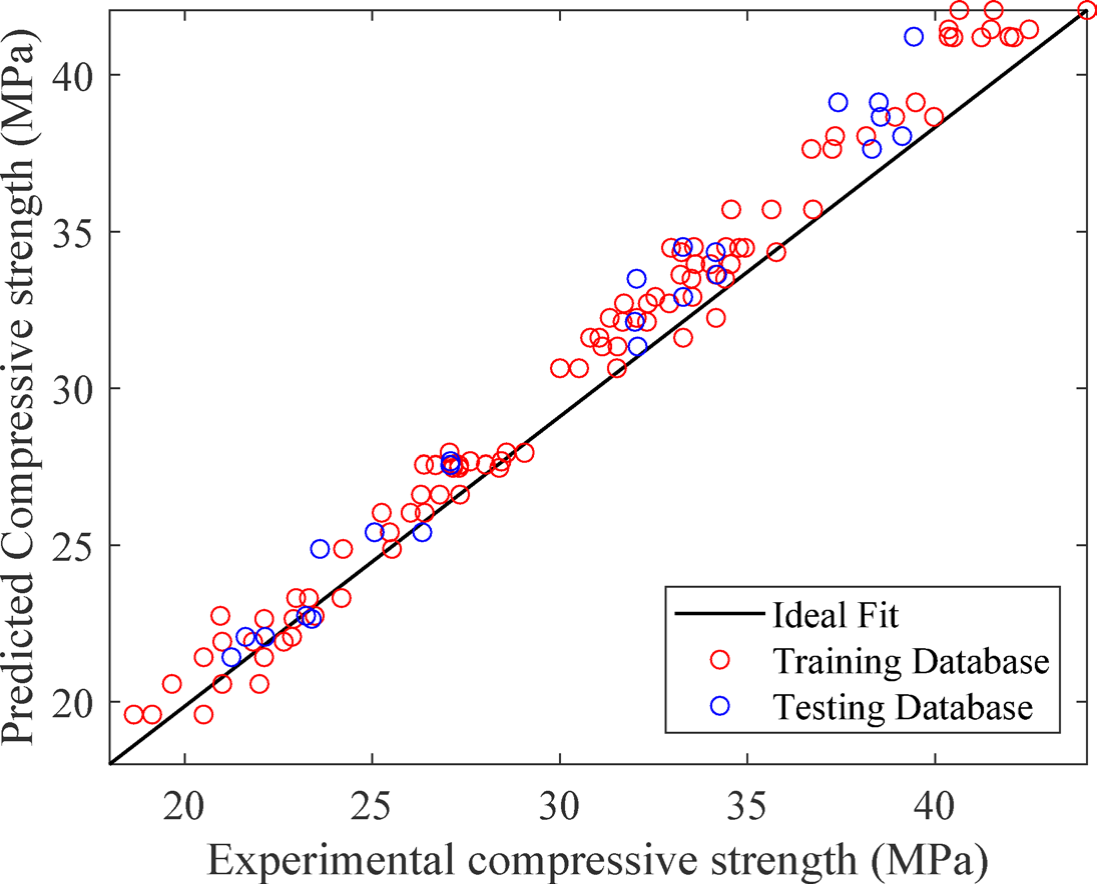
Comparison of experimental and ANN-predicted compressive strength values for training and testing datasets, plotted against the ideal fit line to assess prediction accuracy.

**Fig. 17:**
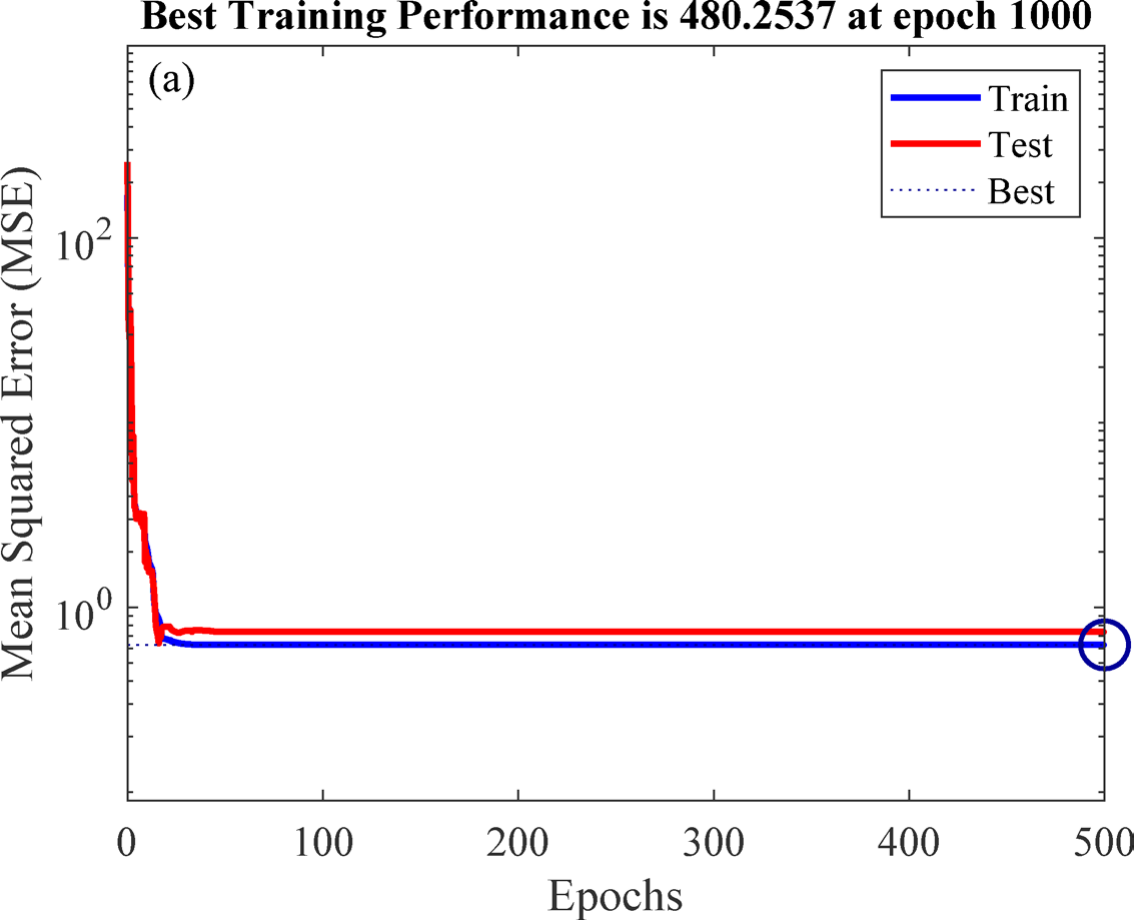
ANN training performance curve showing mean squared error (MSE) variation over epochs for training and testing datasets, with the minimum MSE indicating the best performance.

The Model’s predictive accuracy was further assessed using linear regression, as shown in Fig. 16. This evaluation approach used a graphical representation, with experimental measurements plotted along the abscissa (X-axis) and Model predictions along the ordinate (Y-axis). This conventional machine learning validation technique provides quantitative measures of prediction bias, systematic errors in the model’s output, and the overall correlation between predicted and observed values^103^. The slope coefficient is a critical indicator of model performance; values approaching unity indicate greater predictive fidelity. Clustering data points relative to the regression line in Fig. 16 visually confirms the model’s accuracy. The methodology has been significantly useful in comparative studies analyzing AI-driven slope stability assessments, where regression slopes between predicted and observed values help quantify model accuracy^1,104^. Khan et al^105^. demonstrated that slope-derived metrics could enhance interpretability in neural network-based geotechnical simulations, a technique later adopted in hybrid deep learning frameworks. Statistical analysis reveals strong agreement between the computational model’s predictions and experimental measurements. The calculated slope values, substantially exceeding the established threshold of 0.80, provide quantitative evidence of this robust correlation. These findings indicate the model successfully captures the fundamental relationships governing the system’s behavior. Furthermore, the solution demonstrates reliability across both training and validation phases. The best training performance (480.2537) was achieved at Epoch 1000, as shown in Fig. 17, indicating successful convergence driven by stable learning dynamics and effective backpropagation. This RMSE value represents the model’s minimum training error, indicating its ability to accurately capture data patterns. The gradual error reduction of over 1000 epochs, without premature convergence, confirms the appropriateness of the training parameters and suggests that the model has reached its maximum predictive potential, establishing a reliable foundation for subsequent validation and practical application in predicting the compressive strength of cementitious composites.

**Fig. 18:**
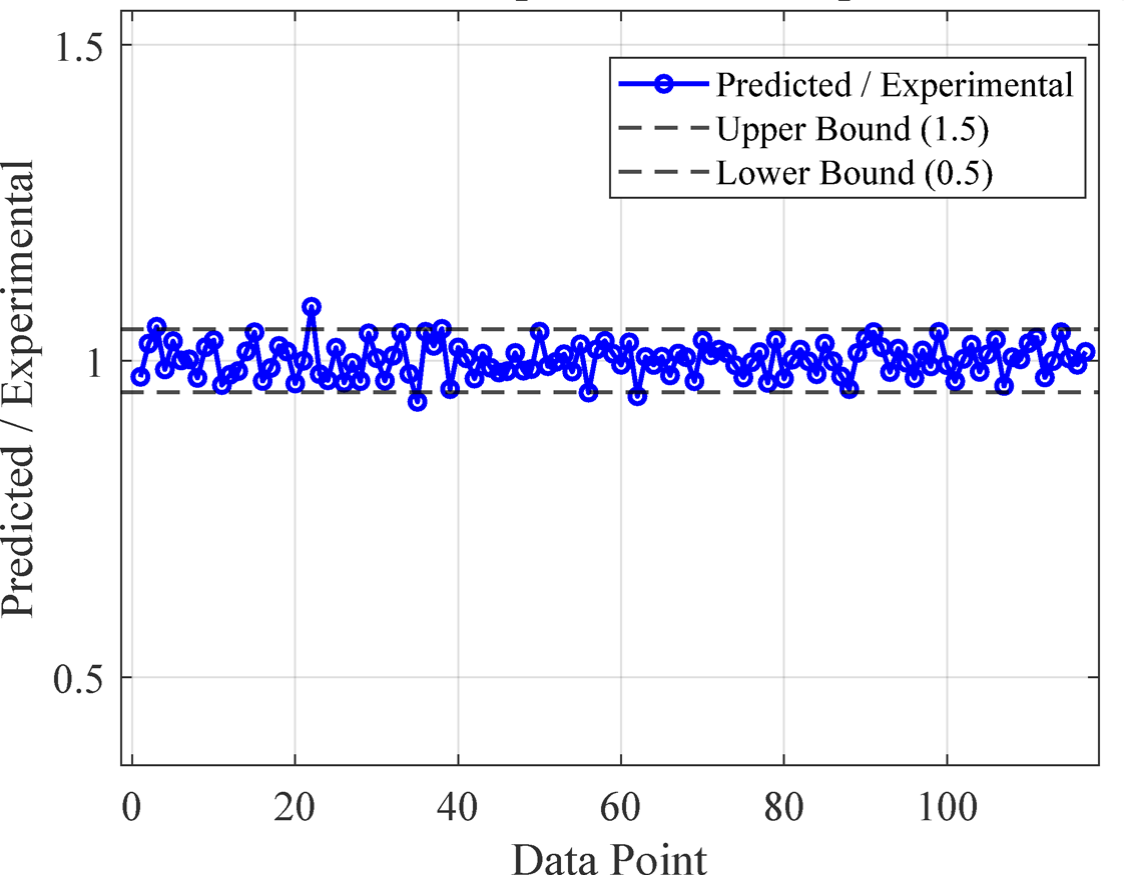
Ratio of predicted to experimental compressive strength.

**Fig. 19:**
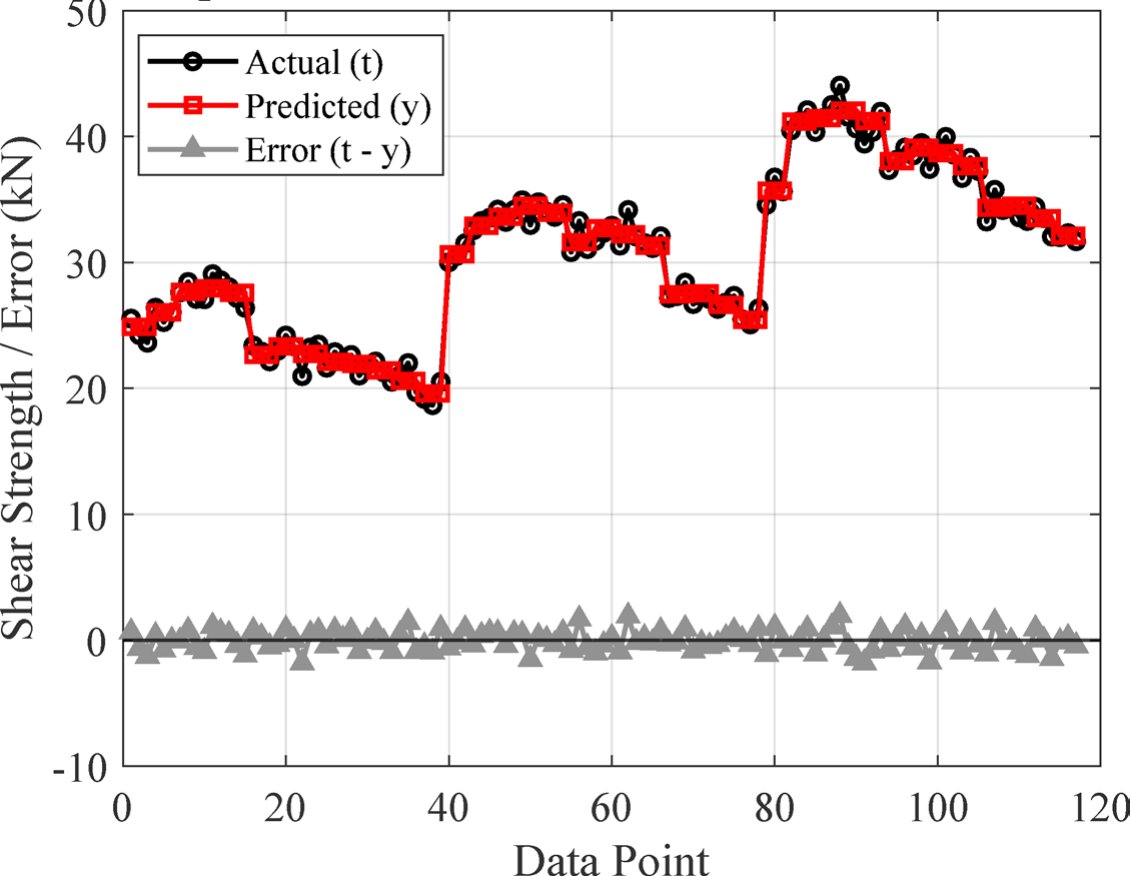
Comparison of actual, predicted, and error values.

**Fig. 20:**
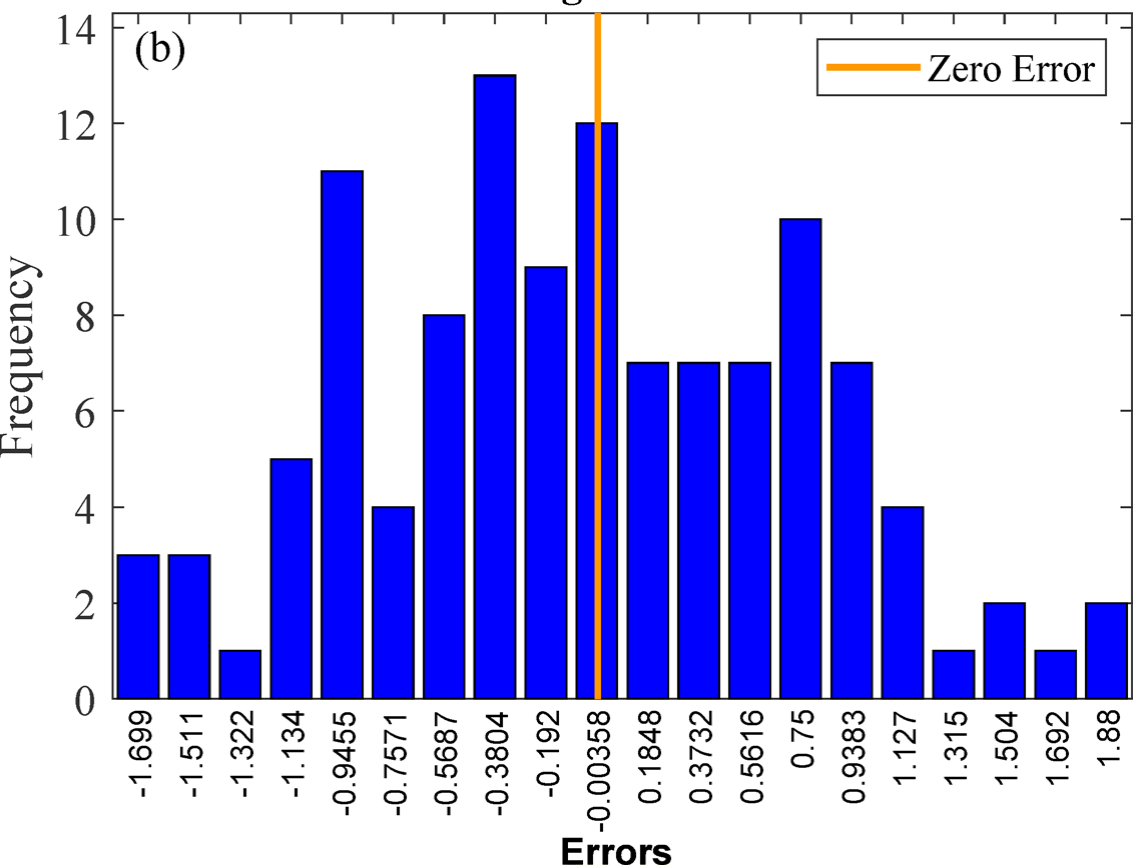
Error histogram of the ANN model using 20 bins, showing the distribution of prediction errors and the zero-error reference line for accuracy evaluation.

The statistical assessment was enhanced through additional quantitative measures, including predicted-to-experimental ratio analysis Fig. 18 and detailed error distribution evaluation Fig. 19. The histogram error reveals exceptional model performance, as shown in Fig. 20. This high-accuracy distribution demonstrates the model’s robust predictive capability. The histogram’s narrow spread particularly highlights the model’s ability to capture the complex, nonlinear behavior of ternary-modified cementitious composites. Such performance metrics surpass typical benchmarks for computational materials science models, suggesting strong potential for practical implementation in mix design optimization and strength prediction applications^106^. The results confirm that the ANN architecture successfully learned the critical parameter relationships governing the development of compressive strength in these advanced ternary-based systems.

The statistical evaluation confirms the ANN model effectively predicts the compressive strength of ternary-modified cementitious composites, demonstrating a strong correlation (*R* > 0.99) between predicted and experimental values with acceptable error margins (MAE < 1 MPa, RMSE < 1 MPa). The regression slope exceeds 0.80, and over 96% of predictions fall within ± 10% of actual measurements, validating the model’s reliability. The model’s consistent performance across both the training and validation datasets also indicates robust generalization. These results establish the ANN as an accurate predictive tool for predicting ternary-based high-strength concrete mixtures in both research and practical applications.

#### Model validation

**Fig. 21:**
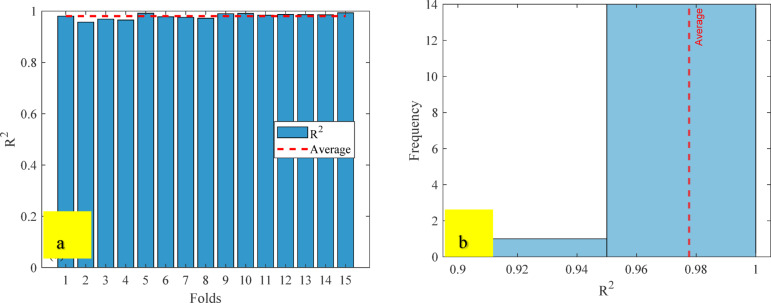
ANN K-fold cross-validation results: **(a)** R² values across individual folds for training and testing datasets, and **(b)** frequency distribution of R² values showing model stability and generalization.

A cross-validation process (K = 15) was conducted to ensure that the proposed ANN model is robust and can be generalized, as shown in Fig. 21. The network architecture was tested on 1 to 10 hidden layers. The results of the performance indices of the training and testing folds are summarized in Table 17. The findings indicate high consistency between the training (R² = 0.985) and testing (R² = 0.981) steps, with only slight differences in RMSE and MAE (< 0.9 MPa). Optimal predictive performance was achieved with a 4-neuron hidden-layer configuration, demonstrating that the model can capture nonlinear relationships between mix constituents and compressive strength without overfitting. The results support the accuracy of the ANN structure and the consistency of its forecasts for EMS-RHA concrete structures.

**Fig. 22:**
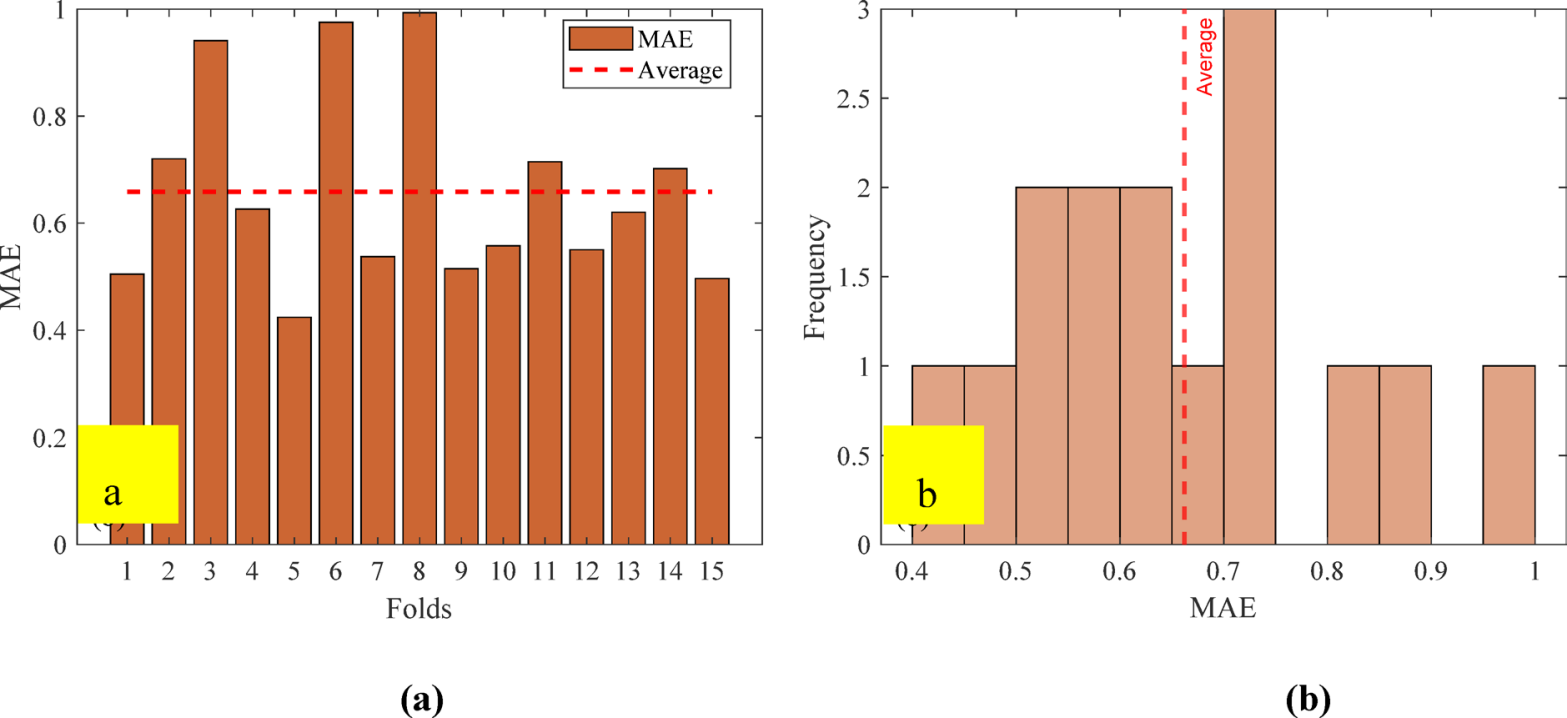
Variation of MAE across K-folds for the ANN model: **(a)** MAE values across folds for training and testing datasets, and **(b)** histogram of MAE distribution with mean value indicated, demonstrating prediction stability.

Figure 22 shows the distribution and variation of the Mean Absolute Error (MAE) obtained in K-fold cross-validation. The fold values of the MAE (Fig. 22 (a)) remain consistently below 1 MPa, indicating stable predictive reliability and low variation between the training and testing subsets. The histograms (Fig. 22 (b)) indicating a narrow, symmetric distribution centered around (MAE = 0.7 MPa), confirming the robustness and reliability of the ANN model in estimating compressive strength without overfitting.

### Comparison of models

The evaluation demonstrates distinct strengths for both modeling approaches: the ANN excels in nonlinear predictive accuracy, while RSM offers superior interpretability of variable interactions. Statistical comparison reveals that the ANN achieves higher correlation coefficients (R² = 0.9851 for training, 0.9811 for validation) than RSM (R² = 0.90), indicating better agreement with the experimental data. Error metrics further confirm the advantage of ANN, showing lower prediction errors (ANN: MAE = 0.650–0.704 MPa, RMSE = 0.793–0.860 MPa) compared to RSM’s performance.

Table 18 presents a comparison of performance metrics (R², MAE, RMSE) for both models. The results align with previous studies, confirming that RSM is used for optimization and ANN for prediction, with both models showing similar predictive accuracy^107–109^. This is expected, as both models aim to accurately model the relationships between the independent variables (EMS and RHA dosages) and the dependent variable (CS). The comparison of these models highlights their effectiveness in both optimization and prediction, demonstrating that both methods provide valuable insights for concrete mix design.

**Table 18:** Performance comparison of ANN and RSM models.

Statistical Index	ANN Model		RSM Model
	Training data	Validation data	
R^2^	0.985	0.981	0.90
RMSE	0.793	0.860	1.73
MAE	0.650	0.704	2.78

The novelty of this research lies in the development of a dual predictive framework combining RSM and ANN. Both models showed strong agreement with experimental data, with ANN offering superior prediction accuracy. The integration of statistical and machine learning techniques provides a robust platform for mix optimization, reducing reliance on trial-and-error in laboratory investigations and enabling accurate predictions of strength development. This strategy offers a practical pathway for the industry to accelerate the adoption of sustainable ternary blends, improving concrete mix designs while minimizing material waste and costs.

## Conclusions

- This study investigated the synergistic effects of extracted micro-silica (EMS) and rice husk ash (RHA) on the mechanical and durability performance of ternary cementitious systems. Concrete mixtures with varying EMS dosages (5%, 10%, 15%) and RHA dosages (5–40%) were evaluated for compressive strength at 14, 28, and 56 days. The results confirmed that the performance of EMS–RHA ternary concrete is highly dosage-dependent. Optimal dosages of EMS (5–10%) and RHA (15–30%) resulted in significant improvements in strength, with mixes E05R15 and E10R25 achieving 11.56% and 5.98% higher compressive strength, respectively, at 28 days, compared to the control mix.
- Conversely, high replacement levels, particularly E15R35, resulted in a 12.59% reduction in strength, highlighting the detrimental effects of over-substitution. Water permeability results further supported the durability benefits of EMS and RHA incorporation at optimized dosages. Mixes E05R15 and E10R25 showed reduced permeability, consistent with their refined microstructure observed in SEM images, whereas E15R35 exhibited higher permeability due to the formation of porous zones and agglomerated micro silica particles.
- SEM analysis further validated these findings, showing that mixtures with optimal dosages (E05R15 and E10R25) had dense C-S-H networks and uniformly dispersed particles, leading to superior performance. However, high-dosage mixes (E15R35) exhibited poor dispersion, micro-silica agglomeration, and interconnected voids, compromising the microstructure and reducing mechanical integrity.
- RSM effectively quantified the influence and interaction of variables, with highly significant Model terms (*p* < 0.001) and strong predictive capability (R² > 0.95). ANN further improved prediction accuracy, achieving R² values above 0.98 with lower RMSE and MAE than RSM, indicating superior predictive accuracy for nonlinear behavior. Optimization results identified promising EMS–RHA replacement ranges that maintain workability while improving strength properties.

Future research should explore long-term durability, microstructural evolution using advanced characterization tools, and scaling the proposed mixture for industrial production. Expanding the modeling framework to include hybrid or deep learning models could further enhance prediction accuracy and optimize multiple performance criteria simultaneously. While this study primarily focuses on the mechanical performance and durability of EMS–RHA ternary concrete, future studies should also include a quantitative analysis of energy and environmental impacts. This could involve assessing CO_2_ emissions reductions and energy consumption associated with using EMS and RHA as partial substitutes for cement in the production of sustainable concrete. Such research would contribute to a more comprehensive understanding of the sustainability of these materials and their potential for sustainable construction.

## Data Availability

The datasets used and/or analyzed during the current study are available from the corresponding author on reasonable request.
